# On the Morphology of Group II Metal Fluoride Nanocrystals at Finite Temperature and Partial Pressure of HF

**DOI:** 10.3390/molecules22040663

**Published:** 2017-04-21

**Authors:** Zeinab Kaawar, Stefan Mahn, Erhard Kemnitz, Beate Paulus

**Affiliations:** 1 Institut für Chemie und Biochemie, Freie Universität Berlin, Takustr. 3, 14195 Berlin, Germany; kaawarzeinab@zedat.fu-berlin.de; 2 Institut für Chemie, Humboldt-Universität zu Berlin, Brook-Taylor-Str. 2, 12489 Berlin, Germany; Stefan.Mahn@t-online.de (S.M.); erhard.kemnitz@chemie.hu-berlin.de (E.K.)

**Keywords:** nanocrystals, DFT calculations, surface thermodynamics, TEM measurements

## Abstract

We have investigated the bulk and surface properties of the group II metal fluorides CaF${}_{2}$, SrF${}_{2}$ and BaF${}_{2}$ using periodic density functional theory (DFT) calculations and surface thermodynamics. Our bulk results show that the best agreement with experiment is achieved with the B3LYP and PBE functionals. We determined the relative importance of the low index surfaces in vacuum and found that an fluoride microcrystal exposes only the (111) surface in which the undercoordinated cations are sevenfold coordinated. With methods of ab initio surface thermodynamics, we analyzed the stability of different surfaces under hydrogen fluoride (HF) pressure and determined the presumable shape of the crystals with respect to different HF concentrations and temperatures. In the case of CaF${}_{2}$ and SrF${}_{2}$, the calculated shapes of the crystals agree well with TEM images of fluorolytic sol-gel synthesized nanocrystals at room temperature and high HF concentration.

## 1. Introduction

During the last several years, alkaline earth metal fluorides have been the subject of many experimental and theoretical studies, due to their high technological importance in different fields. Particularly, they exhibit exceptional optical properties and are considered as excellent candidates for antireflective optical coatings, spectroscopy and laser applications, mainly due to their optical transmittance ranging from low UV up to the high IR region. Because of their low refractive index and their low solubility in water, CaF${}_{2}$ and SrF${}_{2}$ are often used as chemical resistant, weather-proof coating materials for high quality optical windows [1]. Moreover, they have shown to improve the mechanical and chemical properties of dental materials when they are used as inorganic fillers in dental composites [2,3]. BaF${}_{2}$ is considered as the fastest reacting luminescent material [4] and is widely used for γ-ray and elementary particle detection [5]. Whereas, in the former applications, bulk properties are essential, for another promising application, surface properties are crucial. Metal fluorides can be used as catalysts for a great number of reactions [6,7]. The catalytic interest in these materials was renewed by the development of the fluorolytic sol-gel synthesis [8], which starts with a reaction of a metal alkoxide with hydrogen fluoride (HF) in a suitable organic solvent to form a transparent sol and continues with a post-treatment, after which the desired nanomaterial is obtained. Varying the synthetic conditions, such as temperature and the partial pressure of hydrogen fluoride, leads to a change in the morphology of the crystallites and therefore affects the surface properties and hence the catalytic behaviour of the nanomaterials.

Especially on CaF${}_{2},$ a large amount of experimental and theoretical works has been published during the past several years [9,10,11,12,13]. In the beginning of the nineties, Catti et al. [14] calculated the electronic band structure of CaF${}_{2}$ applying ab initio periodic Hartree–Fock calculations. Other works dealing with the electronic structure of CaF${}_{2}$ and BaF${}_{2}$ have been recently published [15,16], in which the bulk and surface electronic structures were calculated from first principles. Shi et al. also calculated surface energies of the (111), (110) and (100) surfaces of CaF${}_{2}$ and BaF${}_{2}$ using the hybrid B3PW functional. The surface structure, as well as the reactivity of CaF${}_{2}$, has been investigated by means of pseudopotential plane-wave calculations where the adsorption of water molecules and methanoic acid is described in detail [17]. Moreover, pressure induced phase transitions of CaF${}_{2}$, SrF${}_{2}$ and BaF${}_{2}$ were studied [18,19], showing that the fluorite structure is the ground state phase for the three investigated materials.

To the best of our knowledge, up to now, there are no published results dealing with the topic of the effect of temperature and pressure of HF on the surface structure and the morphology of nanocrystals of CaF${}_{2}$, SrF${}_{2}$ and BaF${}_{2}$. To understand these materials’ properties better, we investigate in this study the surface structure of the group II metal fluorides CaF${}_{2}$, SrF${}_{2}$ and BaF${}_{2}$. We study the influence of temperature and the partial pressure of HF on the stability of low index MF${}_{2}$ surfaces (M = Ca, Sr or Ba), and we determine the most stable shapes of the crystals under different conditions. TEM images of sol-gel synthesized nanocrystals of CaF${}_{2}$ and SrF${}_{2}$ are used for comparison to computational results.

## 2. Results

### 2.1. Bulk Calculations

As the starting point of our calculations, we have tested how different methods reproduce the experimentally observable bulk properties. We have calculated the lattice constant, the bulk modulus, the cohesive energy and the lattice energy of the three systems CaF${}_{2}$, SrF${}_{2}$ and BaF${}_{2}$. The Hartree–Fock method as well as all DFT functionals used describe these bulk properties reasonably well (see Table 1). Best agreement with experiment [20,21,22,23,24] is achieved at the PBE (Perdew-Burke-Ernzerhof) level that overestimates the lattice constant at most by 0.7% and underestimates the bulk modulus by 10% only. The cohesive energy is slightly overestimated by at most 11% for BaF${}_{2}$ and the lattice energy is underestimated by 3%. Hence, this functional yields reasonable structural bulk properties and is used in the surface calculations.

### 2.2. Clean Surfaces

**Figure 1:**
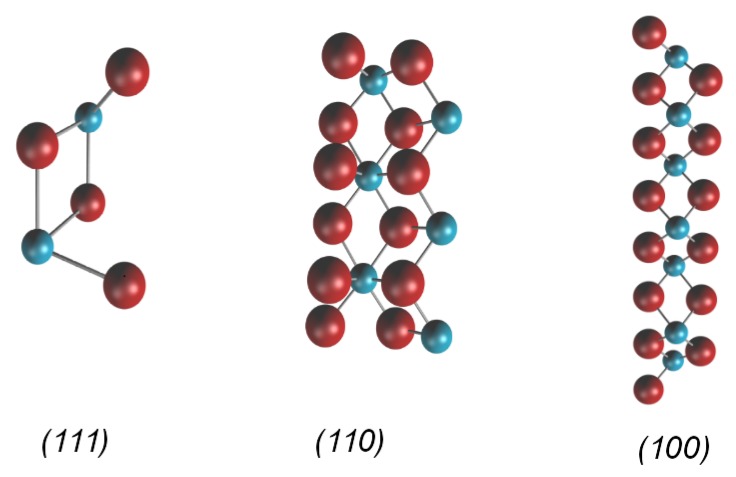
Primitive unit cells of relaxed symmetric slabs of CaF${}_{2}$ surfaces. For the (111) surface, six layers are used, for the (110) surface, six layers, each consisting of a CaF${}_{2}$-unit, and for the (100), 15 layers are used. Fluorides are drawn in red and calcium in blue.

The occurring cleavage plane of a MF${}_{2}$ crystal is the (111) surface, which consists of planes of metal ions in a hexagonal array with a layer of fluoride ions both above and below [25]. The (111) surface is thus terminated with fluoride ions and seven-coordinated metal ions occur. Two other relatively stable low index surfaces of the MF${}_{2}$ crystals are the (110) and the (100) surfaces. We performed periodic slab calculations for the three mentioned surfaces of the materials under study. The slabs were allowed to relax, while the size of the surface unit cell was kept fixed to the bulk value. The relaxed primitive unit cells of the three low index surfaces of CaF${}_{2}$ are shown in Figure 1.

We calculated surface energies using the PBE functional (see Table 2), which allowed us to predict the shape of a MF${}_{2}$ crystal in vacuum using the Wulff procedure [26].

The (111) surface was found to be the most stable one with a surface energy of 0.47 J/m${}^{2}$ for the CaF${}_{2}$ material, which is in very good agreement with the only experimental estimation that, to our knowledge, is available (0.45 J/m${}^{2}$) [27]. The calculated surface energy of the (110) surface is considerably larger (0.71 J/m${}^{2}$), which makes it less stable. The (100) surface was found to be the least stable one (0.95 J/m${}^{2}$). Our results agree well with ab initio Hartree–Fock calculations by Puchin et al. [28] and with earlier theoretical work by Tasker [29] based on an ionic shell model.

The same energetic order was found for SrF${}_{2}$ and BaF${}_{2}$, whose calculated surface energies were lower compared to CaF${}_{2}$ with BaF${}_{2}$ having the lowest values. The calculated surface energies of the (111) surfaces of SrF${}_{2}$ and BaF${}_{2}$ are in good agreement with the experimental cleavage energies measured for the two considered crystals [27,30]. Depending on the cleavage conditions and the applied formulas to determine the energy from the experimentally measured data, the experimental surface energy for (111) SrF${}_{2}$ varies between 0.26 and 0.46 J/m${}^{2}$ [30]. Tasker [29] also calculated the surface energy of the (111) and (110) surfaces of SrF${}_{2}$ and BaF${}_{2}$; their results obtained with an ionic-shell model compare very well with our first priniciple results. Due to the high ionic character of the material, even such a simplified model can reasonably well resemble the interactions on the surfaces.

The Wulff constructions of the three investigated crystals show that, in vacuum, only the (111) surface is exposed; thus, the predicted shape of a MF${}_{2}$ crystal in vacuum is an octahedron.

### 2.3. Crystal Shapes at Thermodynamic Equilibrium

**Figure 2:**
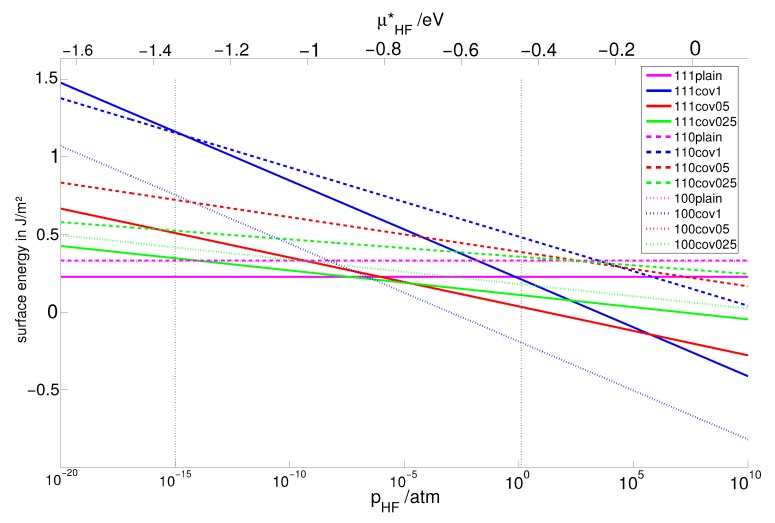
Variation of the surface energy as a function of the pressure of HF for the three low index surfaces of CaF${}_{2}$ at 300 K.

Using the method of ab initio surface thermodynamics, we investigated surface energies as a function of the partial pressure of HF at four temperatures. For each material, we have considered the three low index planes of the fluorite structure, each of them as a plain surface and covered them with 100%, 50% and 25% HF. Figure 2 shows exemplarily the variation of the surface energy as a function of the pressure of HF for CaF${}_{2}$ at 300 K. The surface energy of clean MF${}_{2}$ slabs is independent of temperature and pressure; therefore, it is represented as a straight horizontal line. In general, at very low HF pressure, clean surfaces are most stable. An increase in the pressure of HF leads to stabilization of surfaces covered with HF. However, at high temperatures (T = 600 K), even at a partial HF pressure as high as 1 atm, still clean surfaces are dominant and a much higher pressure would be required to stabilize HF adsorption on the surfaces.

Based on the surface energies of the different planes, we can derive the shape of crystals using the Wulff procedure. We have constructed Wulff plots for MF${}_{2}$ crystals at four different temperatures (150, 300, 450 and 600 K) and four different HF pressures (10${}^{-10}$, 10${}^{-5}$, 1 and 10 atm). In the cubic fluorite structure, termination (100) is equivalent to (010) and (001) and the negative of any of these directions. Therefore, if the (100) surface is exposed in the crystal, six facets that correspond to this termination will occur. Analogously, termination (111) is equivalent to all of its possible negative combinations, so it will occur in eight facets whenever its surface energy allows for its exposure. Due to the high surface energy, the (110) surface does not occur at any investigated conditions.

As already discussed, clean surfaces are favored at high temperatures, which results in a very weak effect of the pressure of HF on the shape of crystals. On the other hand, at low temperature where adsorption structures are stabilized, different crystal shapes are observed with a change in the gas phase, as can be seen from the figures in the following sections. These observations will be discussed in detail for each MF${}_{2}$ crystal separately.

**Figure 3:**
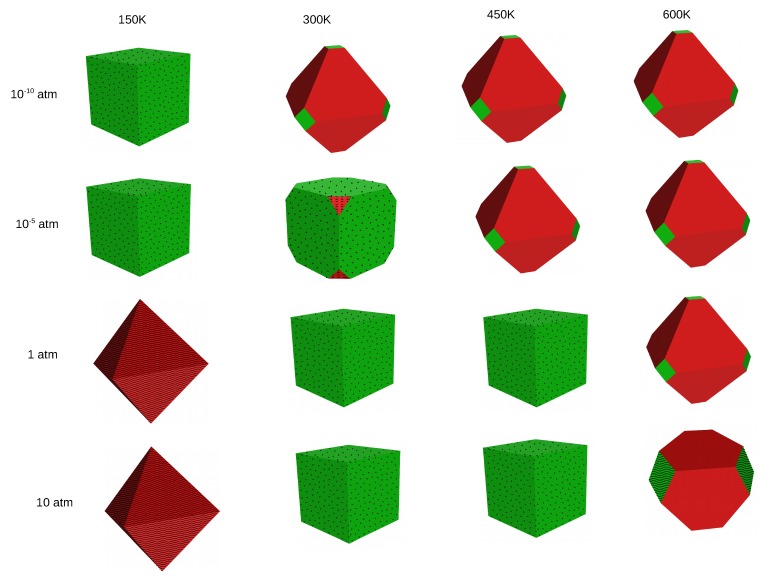
The effect of temperature on the morphology and composition of the CaF${}_{2}$ crystal at four pressure conditions—surface (111) in red and (100) in green. The clean surfaces are indicated by empty planes, the dotted planes correspond to 100% HF coverage, wavy lines to 50% HF coverage and dashed planes to 25% HF coverage.

Figure 3 shows the Wulff plots of CaF${}_{2}$ at different conditions of temperature and pressure. At very low temperature and pressure (T = 150 K and $p_{\mathrm{HF}}$ = 10${}^{-10}$ atm ), the crystal has a cubic shape and only the (100) surface fully covered with HF is exposed. Increasing the temperature at the same pressure leads to transition into an octahedral shape and mainly the (111) clean surface occurs. At even higher temperatures, the shape and composition of the crystal are conserved. At $p_{\mathrm{HF}}$ = 10${}^{-5}$ atm, the same trend is observed, but here the (100) surface with 100% HF coverage remains the main composition of the crystal at T = 300 K, and the (111) with 25% HF coverage shows up in a small percentage at the edges of the cube; a temperature of 450 K is needed to change the shape of the crystal and stabilize only clean surfaces. At standard and high pressures (*p* = 1 atm and *p* = 10 atm), an octahedron exposing only the (111) surface with 50% HF coverage is observed at 150 K, which, upon increase in the temperature up to 300 K, turns out to be a cubic crystal with the (100) surface fully covered, with HF being the unique termination occurring. A further increase in the temperature does not affect the crystal, and a temperature as high as 600 K is necessary to stabilize clean surfaces in an edge-cut octahedron.

**Figure 4:**
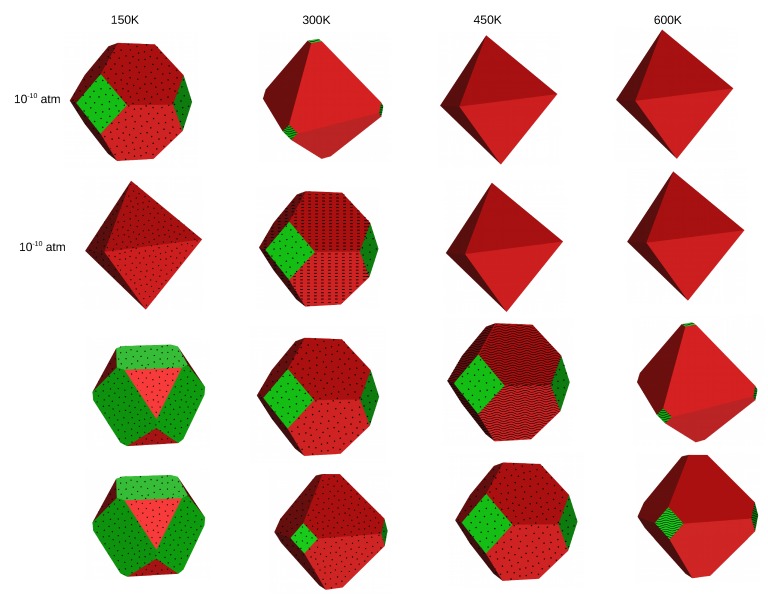
The effect of temperature on the morphology and composition of the SrF${}_{2}$ crystal at four pressure conditions—surface (111) in red and (100) in green. The clean surfaces are indicated by empty planes, the dotted planes correspond to 100% HF coverage, wavy lines to 50% HF coverage and dashed planes to 25% HF coverage.

The Wulff plots of SrF${}_{2}$ are shown in Figure 4. At *p* = 10${}^{-10}$ atm and T = 150 K, the crystal exposes both as a mixture of the (111) and the (100) surfaces, all fully covered with HF. An increase in temperature at constant low pressure stabilizes the (111) clean surface, leading to an octahedral crystal. At 10${}^{-5}$ atm, the crystal exposes fully HF covered (111) surfaces at low temperatures, and the coverage of the (111) surface is reduced to 25% coverage for T = 300 K. Additionally, small contributions of the (100) surface in full coverage occur. Higher temperatures stabilize the clean (111) surface. At standard and high pressure, adsorption is dominant on the surfaces up to a temperature of 450 K, with the (100) contributing more to the crystal shape than the (111) at 150 K, both occurring in full HF coverage, whereas at temperatures higher than 150 K, the (111) surface occurs at a higher percentage. At 600 K, the (111) clean surface is stabilized, with a tiny contribution of the (100) in half coverage.

**Figure 5:**
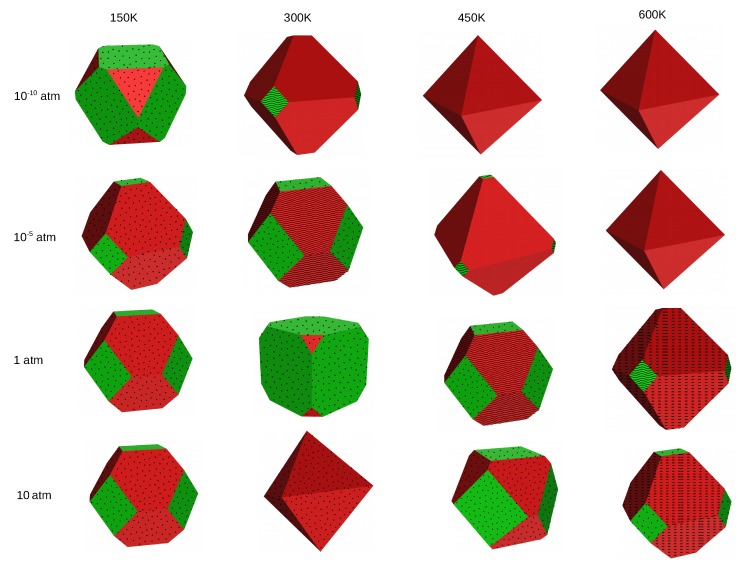
The effect of temperature on the morphology and composition of the BaF${}_{2}$ crystal at four pressure conditions—surface (111) in red and (100) in green. The clean surfaces are indicated by empty planes, the dotted planes correspond to 100% HF coverage, wavy lines to 50% HF coverage and dashed planes to 25% HF coverage.

We present in Figure 5 the Wulff plots of BaF${}_{2}$. At very low pressure ($p_{\mathrm{HF}}$ = 10${}^{-10}$ atm), HF adsorption is dominant on the surfaces only at low temperature (T = 150 K) and an increase in the temperature leads to stabilization of the (111) clean surface. The same trend is observed at $p_{\mathrm{HF}}$ = 10${}^{-5}$ atm, but here we need a temperature higher than 300 K to stabilize the clean (111) surface. At standard and high pressures, HF adsorption is dominant on the surfaces even at a temperature as high as 600 K. The shape and the composition of the crystals at different temperatures are similar for the two pressure conditions, except at T = 300 K, where the crystal has almost a cubic shape and is mainly composed of the fully covered (100) surface at 1 atm, whereas it is an octahedron exposing only the fully covered (111) surface at 10 atm.

In general, all three materials expose clean surfaces at high temperature and surfaces covered with HF at low temperature. When the (111) surface is mainly exposed, the shape of the crystals is octahedral, whereas it is cubic if mainly the (100) surface is exposed. The crystal shapes of SrF${}_{2}$ and BaF${}_{2}$ look similar at most of the conditions, except at low temperature (150 K) and at standard conditions (T = 300 K and $p_{\mathrm{HF}}$ = 1 atm) where they differ in composition of the two surfaces and therefore in shape. The CaF${}_{2}$ crystal differs from the two others, in shape as well as in composition, at several specific conditions. At low temperature and low pressure, while SrF${}_{2}$ and BaF${}_{2}$ are mixtures of the fully HF covered (111) and (100) surfaces, CaF${}_{2}$ is a cubic crystal composed purely of the fully covered (100) surface. The same difference is also observed at T = 300 K and 450 K for $p_{\mathrm{HF}}$ = 1 atm and 10 atm.

### 2.4. Comparison Experimental Found Crystal Shapes

**Figure 6:**
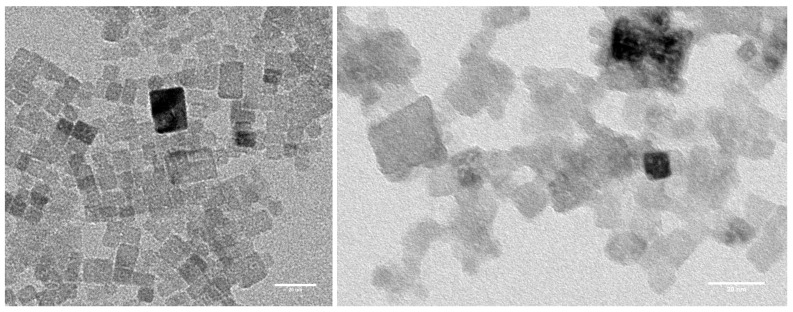
TEM images of CaF${}_{2}$ (**left**) and SrF${}_{2}$ (**right**) nanocrystals.

CaF${}_{2}$ and SrF${}_{2}$ nanocrystals have been synthesized at room temperature under large excess of HF using the fluorolytic sol-gel method. TEM images of the obtained clear sol are shown in Figure 6. In both cases, the size of the nanocrystals is between 5 and 20 nm. In the case of CaF${}_{2}$, a cubic shape of all nanocrystals is clearly seen, which is in agreement with the theoretical prediction of the cubic shape in a large temperature range around room temperature at high excess of HF. For SrF${}_{2}$, the interpretation of TEM images is not so obvious. If one assumes random orientation of the nanocrystals on the substrate, the theoretically predicted octahedral shape would result in a wide distribution of occurring shapes depending on the facet the nanocrystal is lying on. Non-rectangular motifs occur as seen, for example, for the particle in the rightmost lower corner of the TEM image. Most of the exposed nanocrystals for SrF${}_{2}$ look spherically, indicating that different surface cuts are present. This analysis is true for the light grey nanoparticles in the TEM image of SrF${}_{2}$, whereas for the one dark gray nanoparticle, the three-dimensional shape can not be predicted. It can be a cube or an octahedron, looking at the square base plane of the octahedron. In general, the TEM images support the theoretical findings, and CaF${}_{2}$ nanocrystals have a distinct cubic form at room temperature and high excess of HF, whereas SrF${}_{2}$ nanocrystals under the same conditions show a more spherical form, which can be idealized with the theoretical obtained octahedral shape.

We believe that the synthesis of the nanocrystals is determined mainly by thermodynamics because lactic acid as a strongly polar solvent increases the solubility of our lactate precursors. Fluorination by hydrofluoric acid also a strong polarized molecule, and then establishes the ideal thermodynamic prerequisites for a crystallization of nearly insoluble CaF${}_{2}$ and SrF${}_{2}$ nanoparticles in their thermodynamical stable form. For a more unpolar solvent, e.g., ethanol, we could obtain more spherical nanoparticles, which most probably are determined by kinetics.

## 3. Material and Methods

### 3.1. Computational Details

The investigated group II metal fluorides are highly ionic insulators that crystallize in the cubic fluorite structure of space group Fm3m with three ions per unit cell, where each cation is surrounded by eight fluoride ions, which are, in turn, coordinated to four metal ions in a tetrahedral arrangement [31]. All periodic calculations were performed using the CRYSTAL09/13 program package (Universtiy of Torino, Torino, Italy) [32,33]. For the bulk calculations, we employed the Hartree–Fock method and density functional theory (DFT) with five different functionals. These include one functional based on the local density approximation (LDA) [34,35,36], two functionals based on the generalized gradient approximation (GGA) PBE [37] and PW91 [38] and two hybrid functionals, B3LYP [39,40] and B3PW [38,39]. The bulk modulus is obtained by scanning volumes in the range of ± 0.1 around the experimental volume and fitting them to the Birch–Murnagham equation of state (EOS). The surface calculations were performed at the PBE level, adopting the lattice constants and cell parameters obtained from full optimization of the bulk. A stoechiometric symmetric on the surface cut with an inversion center or a mirror plane in the center of slabs consisting of two, six and seven formula units (correspond to six, six and fifteen layers) were employed for the (111), (110) and (100) surfaces, respectively, yielding both a converged surface energy and converged Mulliken charges in the center of the slabs. The data for the convergence with slab thickness are provided in Table S2 in the Supplementary Information.

Different terminations of the surfaces have been regarded. Due to the highly ionic character of the material, only terminations with stoechometric or nearly stoechometric distributions form stable surfaces. In the case of the (100) surface, the stoechiometry is obtained by removing two F${}^{-}$ anions, one from the top and one from the bottom of the slab yielding the most stable termination for this surface. The surface energies are calculated as

(1) $$E_{\mathrm{surf}}=\frac{1}{2A}\left(E_{\mathrm{slab}}-nE_{\mathrm{bulk}}\right),$$

where *n* is the number of MF${}_{2}$ units in the slab, $E_{\mathrm{slab}}$ is the total energy of the relaxed slab, $E_{\mathrm{bulk}}$ is the total energy per bulk unit cell and A is the area of the slab unit cell. The factor $\frac{1}{2}$ is necessary to account for the upper and lower surfaces in a two-dimensional slab.

The F${}^{-}$ anion is described with a valence triple-zeta basis set augmented by a d polarization function (exponent 0.7) [41], previously optimized for the MgF${}_{2}$ solid. The metal cations (Ca${}^{2+}$, Sr${}^{2+}$ and Ba${}^{2+}$) are described with a cc-pVDZ basis set optimized previously in our group [42], where only 10 valence electrons are treated explicitly and the core electrons are simulated by energy-consistent scalar-relativistic pseudopotentials [43]. For the hydrogen atom, we used a VTZ basis set from Bailey et al. [44] developed for HF adsorption on AlF${}_{3}$. We employed shrinking factors of 8 and 16 for the Monkhorst–Pack and Gilat k-point net, respectively. The accuracy of the Coulomb and exchange series is controlled by cutoff parameters ITOL1-5 of 6, 6 ,6, 12, 30 and the total energy convergence criterion is set to 10${}^{-8}$ E${}_{h}$.

With the help of quantum chemical codes for periodic systems, ab initio surface thermodynamics [45] are employed to link results from ab initio calculations to macroscopic properties and predict the stability of surfaces under different conditions of temperature and pressure.

The Gibbs surface energy γ of a solid in thermodynamic equilibrium with a vapor phase of components *i* is defined as

(2) $$\gamma =\frac{1}{2A}\left(G_{slab}-\sum_{i}N_{i}\mu_{i}\right),$$

where $G_{slab}$ is the Gibbs free energy of the slab and A its surface area (the factor 2 accounts for both faces of the slab). $N_{i}$ is the number of species present in the system and $\mu_{i}$ is the chemical potential of the various species *i* and is expressed through thermodynamic equations for the Gibbs energy, but has to be referred to the quantum chemical energy of the species it is applied for:

(3) $$\mu_{i}\left(p_{i},T\right)=E_{i,DFT}+\Delta \mu_{i}^{p^{0}}\left(T\right)+kT\ln\frac{p_{i}}{p^{0}}.$$

The first term in Equation (3) refers to the electronic DFT energy of species *i*. The second term contains all temperature dependent free energy contributions, and it is calculated using tabulated enthalpy and entropy values at standard pressure $p^{0}$ = 1 atm [46,47]. The third term includes the pressure dependence of the chemical potential. For the derivation of the method, we refer to the original literature [45] and to selected applications to fluorides [44] and oxides [48]. We follow closely the procedure described for rutile MgF${}_{2}$ surfaces under HF pressure [49].

### 3.2. Synthesis of Nanoparticles

The synthetic route to both CaF${}_{2}$ and SrF${}_{2}$ nanoparticles was the same. In the following, we present the details for CaF${}_{2}$. To obtain 50 mL of a 1 M CaF${}_{2}$-sol, pre-dried calcium lactate Ca(C${}_{3}$H${}_{5}$O${}_{3}$)${}_{2}$×0.424 H${}_{2}$O (1 eq., 50 mmol, 11.29 g) was dissolved in a mixture of 46 mL lactic acid (85% fcc, Product Number: W261106, Batch Number: MKBT7221V, Sigma-Aldrich Chemie GmbH, Steinheim, Germany) and 1.5 mL formic acid (puriss p.a. 98%, Fluka, LOT SZE90980, Sigma-Aldrich Chemie GmbH, Steinheim, Germany). Constant and powerful stirring was necessary to get a homogenous precursor suspension. For the fluorination step, we used a 39.24 M aqueous hydrogen fluoride solution (2 eq., 100 mmol, 2.55 mL). The reaction seemed to be done after 30 s, but through a lot of small bubbles in the CaF${}_{2}$-sol, we had to stop the stirring barr for 2–3 min. We obtained a clear 1 M CaF${}_{2}$-sol when the bubbles disappeared.

## 4. Conclusions

Alkaline earth metal fluorides (CaF${}_{2}$, SrF${}_{2}$ and BaF${}_{2}$) nanocrystals have been investigated both with computational DFT-based methods and with TEM characterization. We determined the relative importance of the low index surfaces in vacuum and found that a MF${}_{2}$ microcrystal exposes only the (111) surface in which the undercoordinated cations are sevenfold coordinated. With methods of ab initio surface thermodynamics, we analyzed the stability of different surfaces under HF pressure and determined the shape of the nanocrystals under HF concentrations and temperature. All three materials expose clean surfaces at high temperature and surfaces covered with HF at low temperature. Only the (111) and (100) surfaces occur under varying temperature and HF pressure conditions. The ratio between the occurrence of these two surface cuts determines the shape of the nanocrystals. At room temperature and high excess of HF, CaF${}_{2}$ shows cubic nanocrystals, whereas for SrF${}_{2}$ and BaF${}_{2},$ mainly octrahedral nanocrystals are observed. These theoretical findings agree well with the shape of flourolytic sol-gel synthesized nanocrystals of CaF${}_{2}$ and SrF${}_{2}$ characterized with TEM.

## Figures and Tables

**Table 1:** Optimized lattice constant a${}_{0}$ (Å), bulk modulus *B* (GPa), cohesive energy E${}_{coh}$ (eV) and lattice energy E${}_{lat}$ (eV) for CaF${}_{2}$, SrF${}_{2}$ and BaF${}_{2}$ calculated at the PBE level. The energy values are counterpoise corrected. Experimental values are given for comparison.

	CaF${}_{2}$		SrF${}_{2}$		BaF${}_{2}$	
	Calc.	Exp.	calc.	Exp.	Calc.	Exp.
a (Å)	5.50	5.46	5.84	5.8	6.24	6.20
B (GPa)	82	83	62	69	62	57
E${}_{coh}$ (eV)	−16.88	−16.08	−17.01	−15.95	−17.90	−16.01
E${}_{lat}$ (eV)	−26.89	−27.46	−25.35	−26.03	−23.90	−24.58

**Table 2:** PBE-calculated vacuum surface energies for the low-index surfaces of CaF${}_{2}$, SrF${}_{2}$ and BaF${}_{2}$.

Surface	Surface Energy in (J/m${}^{2}$)		
	CaF${}_{2}$	SrF${}_{2}$	BaF${}_{2}$
111	0.47	0.45	0.39
110	0.71	0.67	0.57
100	0.95	0.98	0.86

## Supplementary Materials

The following materials are available online. Calculated bulk properties with different DFT functionals and at the Hartree–Fock level (Table S1); Convergence study of the surface energies with slab thickness (Table S2); Variation of the surface energy as a function of the pressure of HF for the three low index surfaces of MF${}_{2}$ at 150 K, 300 K, 450 K and 600 K respectively (Figures S1–S11; atomic coordinates of all relaxed slabs).
