# Supplementary material for: On the Morphology of Group II Metal Fluoride Nanocrystals at Finite Temperature and Partial Pressure of HF
Source: Molecules. 2017 Apr 21;22(4):663. doi: 10.3390/molecules22040663 (PMC6154342; doi:10.3390/molecules22040663)
Supplement: Supplementary file 1 [file molecules-22-00663-s001.pdf]

MDPI

Article

Supplementary Material: On the morphology of group II metal fluoride nanocrystals at finite temperature and partial pressure of HF

Zeinab Kaawar [1], Stefan Mahn [2], Erhard Kemnitz [2] and Beate Paulus [1]*

[1] Institut für Chemie und Biochemie, Freie Universität Berlin, Takustr. 3, 14195 Berlin, Germany;
[2] Institut für Chemie, Humboldt-Universität zu Berlin, Brook-Taylor-Str. 2, 12489 Berlin, Germany

Academic Editor: Arnaud Gautier
Received: 2 February 2017; Accepted: 14 April 2017; Published: date

1. Bulk parameters with Hartree-Fock and different DFT functionals

Table S1. Optimized bulk parameters for CaF_2, SrF_2 and BaF_2 using Hartree-Fock and different DFT functionals.

		a (Å)	B (GPa)	E_{coh} (eV)	E_{lat} (eV)
CaF_2	Expt.	5.463	82.71	-16.08	-27.46
	HF	5.509	87.01	-13.94	-26.83
	B3LYP	5.496	85.54	-16.44	-26.90
	B3PW	5.475	86.91	-16.34	-26.71
	LDA	5.328	85.14	-18.88	-28.42
	PBE	5.501	82.23	-16.88	-26.89
	PWGGA	5.491	84.03	-17.03	-26.99
SrF_2	Expt.	5.799	69	-15.95	-26.03
	HF	5.869	65.91	-14.30	-25.30
	B3LYP	5.847	63.09	-16.63	-25.33
	B3PW	5.814	68.08	-16.51	-25.20
	LDA	5.670	88.72	-18.90	-26.81
	PBE	5.843	61.91	-17.01	-25.35
	PWGGA	5.830	61.39	-17.18	-25.44
BaF_2	Expt.	6.20	57	-16.01	-24.58
	HF	6.314	53.87	-15.10	-23.66
	B3LYP	6.260	61.25	-17.53	-23.79
	B3PW	6.224	64.94	-17.38	-23.73
	LDA	6.061	73.34	-19.73	-25.31
	PBE	6.240	62.36	-17.90	-23.90
	PWGGA	6.230	63.49	-18.07	-24.00

2. Convergence of surface energies with slab thickness

Symmetric slabs with a central reflection plane are constructed for each surface. The convergence of the surface energy with the number of layers in the slab is tested and presented in Tab. S2. A layer is defined as different z-coordinate (z axis perpendicular to the surface). For the (111) surface each atom is in its own layer. For the (110) surface a CaF_2-unit is forming a layer and for the (100) surface Ca has it own layer, whereas two fluorides form one joint layer.

Table S2. Surface energies γ (J/m^2) for the three stable surfaces of CaF_2, SrF_2 and BaF_2 at different slab thicknesses.

CaF_2			SrF_2			BaF_2		
(hkl)	Nb of layers	γ	(hkl)	Nb of layers	γ	(hkl)	Nb of layers	γ
(111)	6	0.470	(111)	6	0.451	(111)	6	0.386
	9	0.472		9	0.454		9	0.388
	12	0.472		12	0.454		12	0.388
(110)	5	0.723	(110)	5	0.681	(110)	5	0.572
	6	0.714		6	0.675		6	0.568
	7	0.718		7	0.677		7	0.569
(100)	13	0.944	(100)	13	0.981	(100)	13	0.860
	15	0.955		15	0.984		15	0.859
	17	0.951		17	0.985		17	0.859

3. Surface energies as function of the pressure

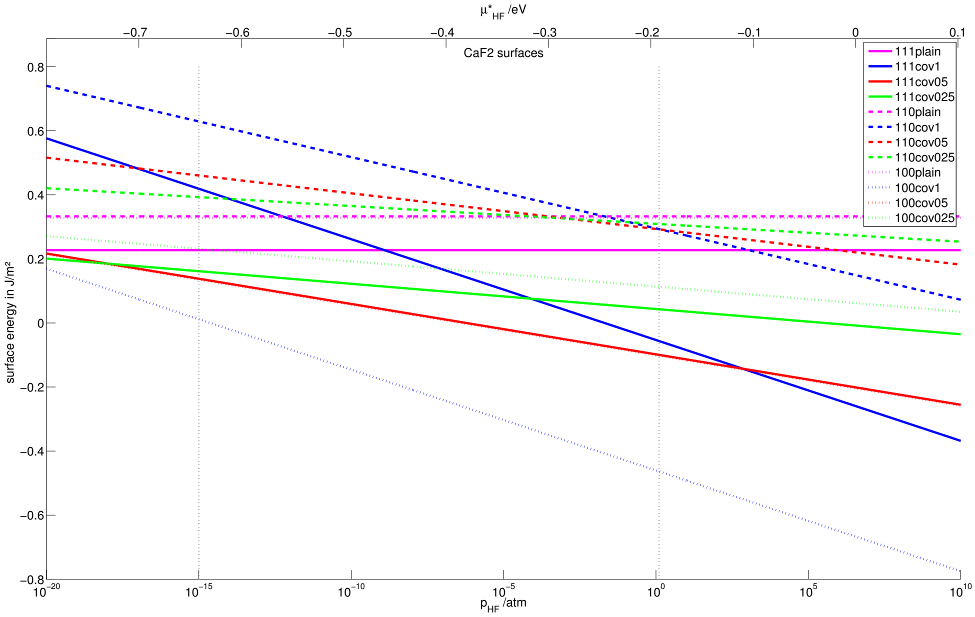

Figure S1. Variation of the surface energy as a function of the pressure of HF for the three low index surfaces of CaF_2 at 150 K.

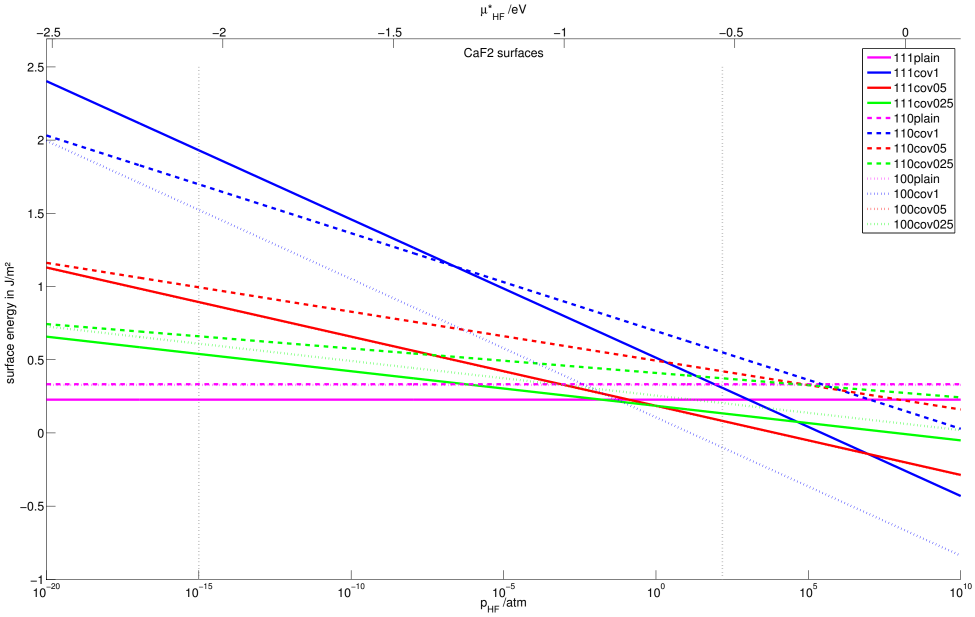

Figure S2. Variation of the surface energy as a function of the pressure of HF for the three low index surfaces of CaF_2 at 450 K.

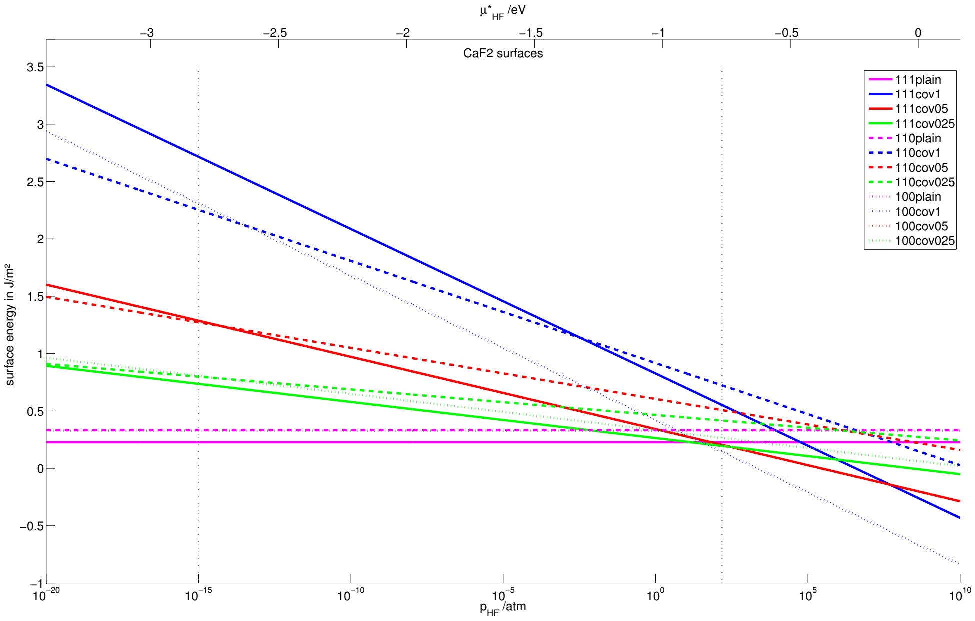

Figure S3. Variation of the surface energy as a function of the pressure of HF for the three low index surfaces of CaF_2 at 600 K.

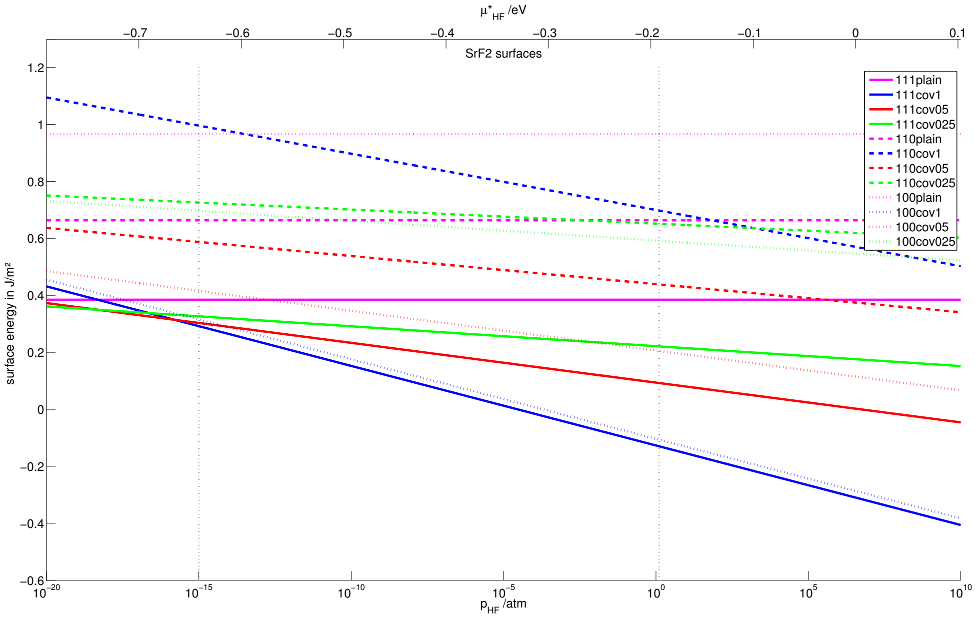

Figure S4. Variation of the surface energy as a function of the pressure of HF for the three low index surfaces of SrF_2 at 150 K.

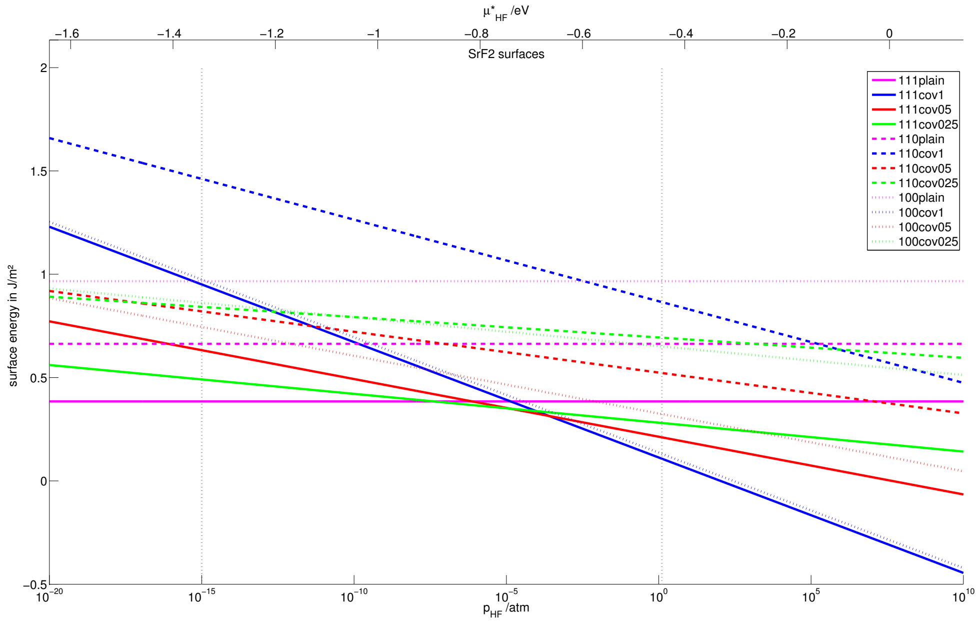

Figure S5. Variation of the surface energy as a function of the pressure of HF for the three low index surfaces of SrF_2 at 300 K.

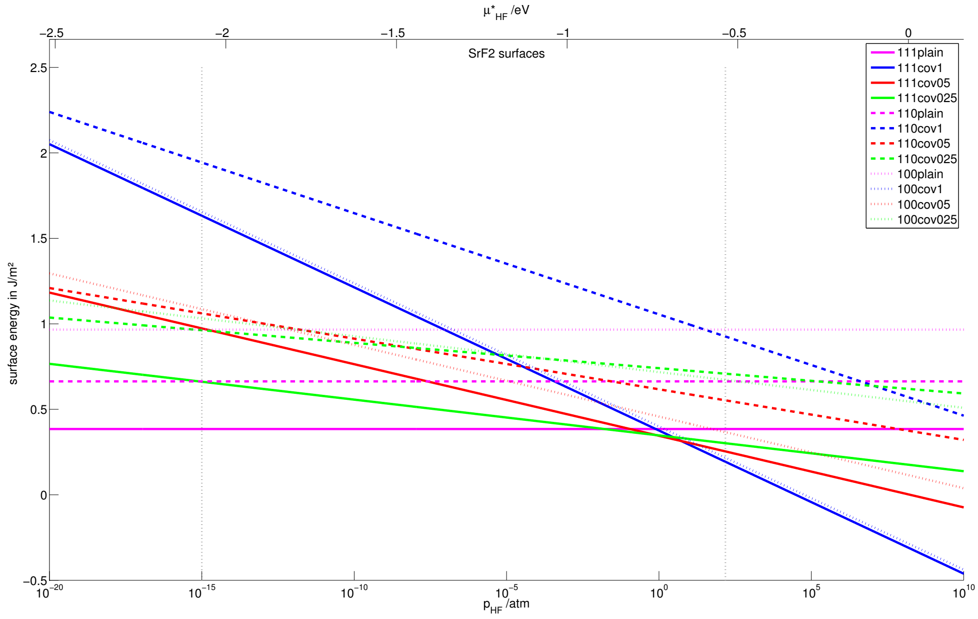

Figure S6. Variation of the surface energy as a function of the pressure of HF for the three low index surfaces of SrF_2 at 450 K.

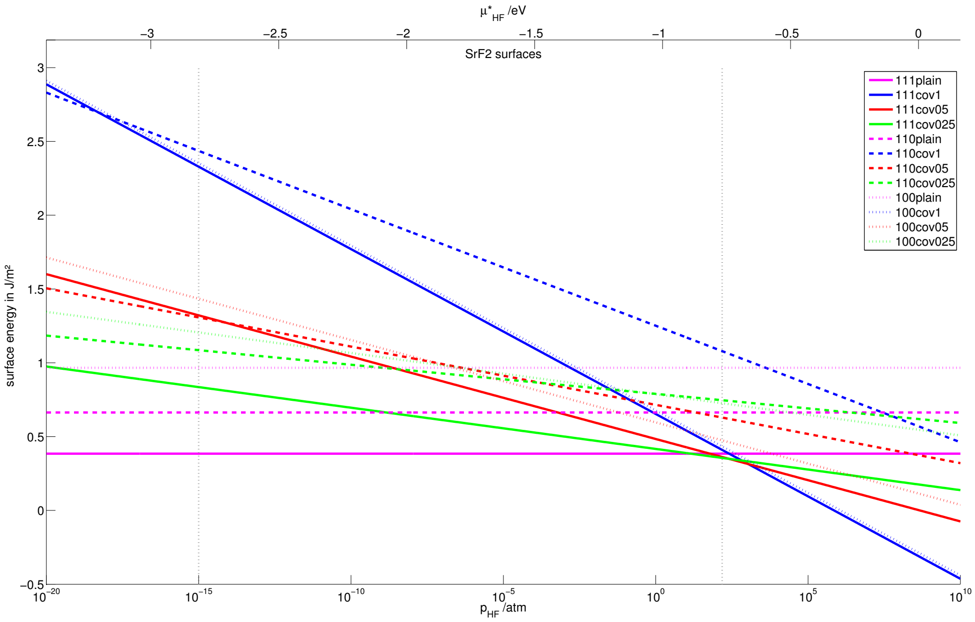

Figure S7. Variation of the surface energy as a function of the pressure of HF for the three low index surfaces of SrF_2 at 600 K.

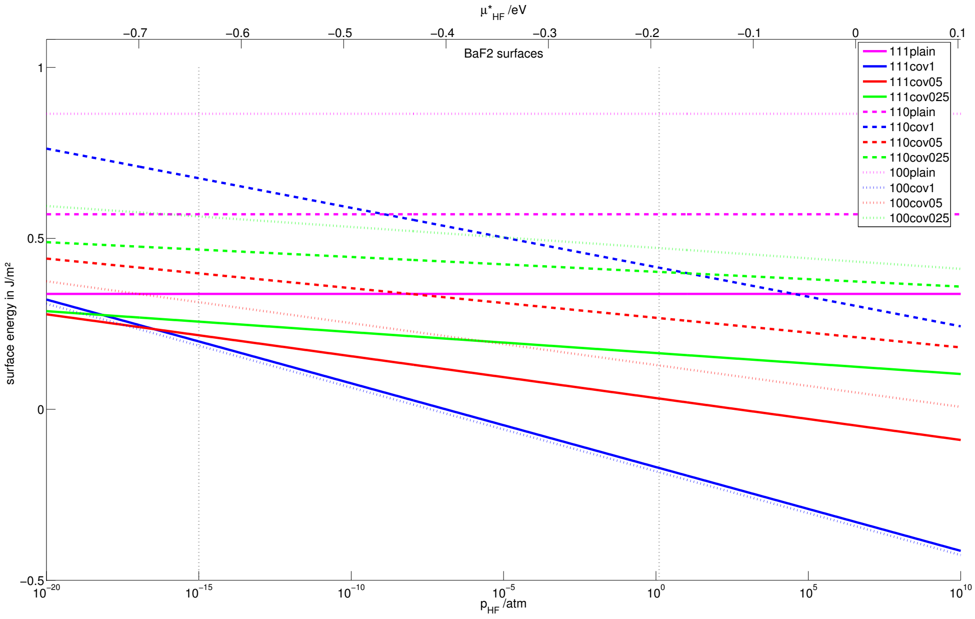

Figure S8. Variation of the surface energy as a function of the pressure of HF for the three low index surfaces of BaF_2 at 150 K.

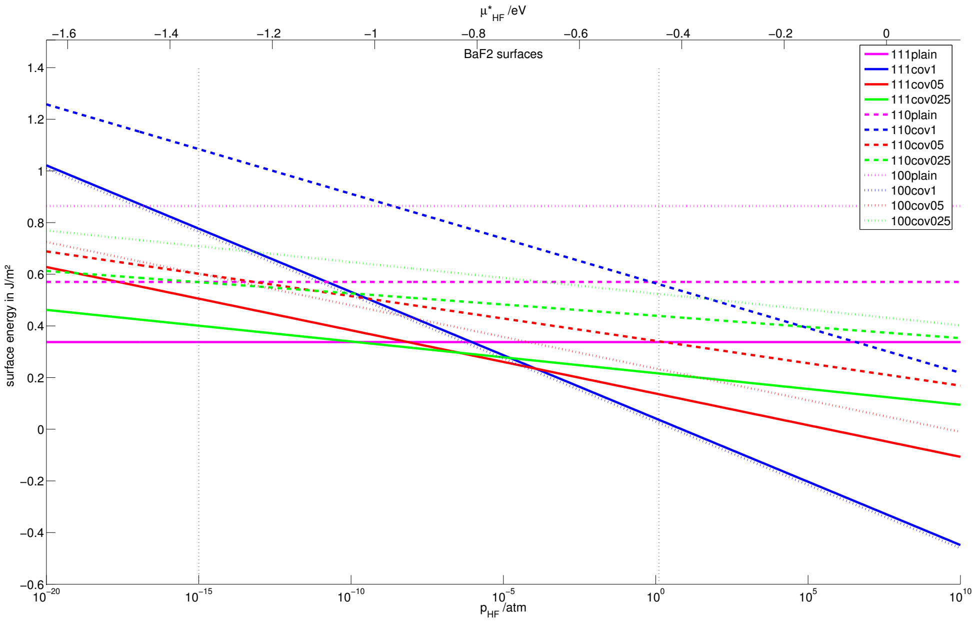

Figure S9. Variation of the surface energy as a function of the pressure of HF for the three low index surfaces of BaF_2 at 300 K.

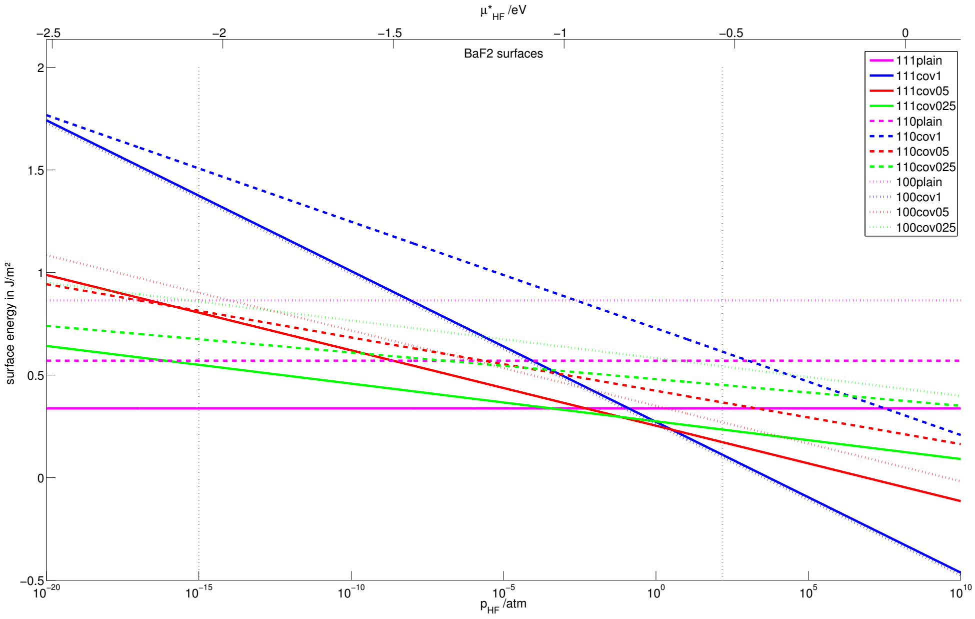

Figure S10. Variation of the surface energy as a function of the pressure of HF for the three low index surfaces of BaF_2 at 450 K.

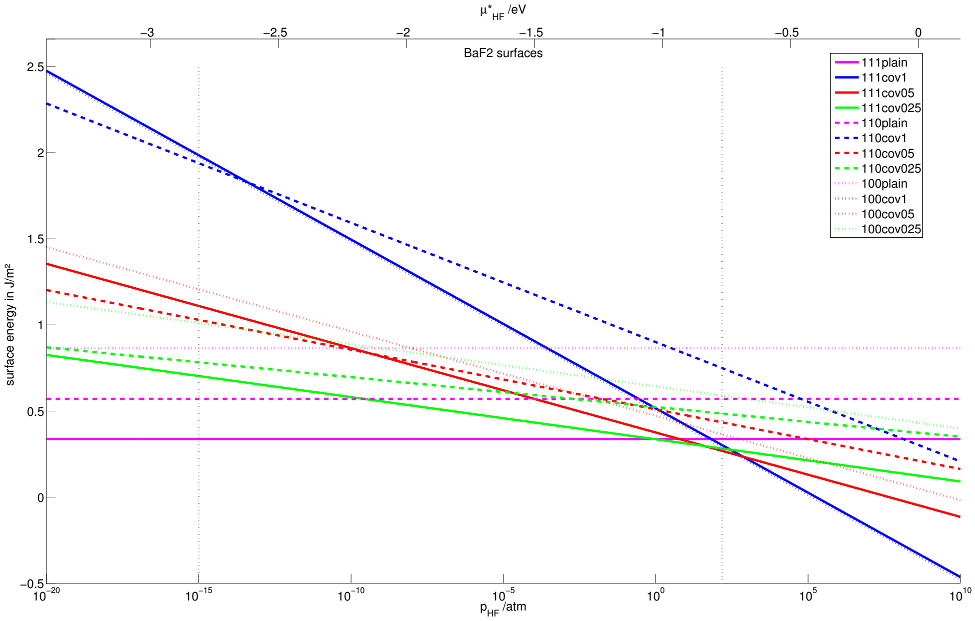

Figure S11. Variation of the surface energy as a function of the pressure of HF for the three low index surfaces of BaF_2 at 600 K.

© 2016 by the authors; licensee MDPI, Basel, Switzerland. This article is an open access article distributed under the terms and conditions of the Creative Commons Attribution (CC-BY) license (http://creativecommons.org/licenses/by/4.0/).

1. CaF_2:

(111) surface, 100% coverage

1	9	F	9.728321703337E-01	-1.690678081025E+00	2.370862283267E+00
2	9	F	-9.728321703337E-01	1.690678081025E+00	-2.370862283267E+00
3	220	CA	-9.729787924481E-01	-5.571628753158E-01	1.580004579276E+00
4	220	CA	9.729787924481E-01	5.571628753158E-01	-1.580004579276E+00
5	9	F	9.732923590636E-01	5.618439802655E-01	7.762492940912E-01
6	9	F	-9.732923590636E-01	-5.618439802655E-01	-7.762492940912E-01
7	109	F	-1.254748082362E+00	-5.604086033089E-01	4.242510948587E+00
8	109	F	1.254748082362E+00	5.604086033089E-01	-4.242510948587E+00
9	1	H	-1.928638566151E+00	-1.211670906084E+00	4.418132538676E+00
10	1	H	1.928638566151E+00	1.211670906084E+00	-4.418132538676E+00

(111) surface, 50% coverage

1	9	F	7.788128108513E-02	6.056354242180E-02	2.253572277919E+00
2	9	F	-7.788128108513E-02	-6.056354242179E-02	-2.253572277919E+00
3	9	F	3.447090306015E+00	-3.125677040127E-01	2.672905786883E+00
4	9	F	-3.447090306015E+00	3.125677040127E-01	-2.672905786883E+00
5	220	CA	1.376965191417E-01	-2.154852767347E+00	1.683131420072E+00
6	220	CA	-1.376965191417E-01	2.154852767347E+00	-1.683131420072E+00
7	220	CA	1.916592293146E+00	1.083902142328E+00	1.506088210881E+00
8	220	CA	-1.916592293146E+00	-1.083902142328E+00	-1.506088210881E+00
9	9	F	5.416516341449E-02	2.271167064005E+00	6.165910509208E-01
10	9	F	-5.416516341449E-02	-2.271167064005E+00	-6.165910509208E-01
11	9	F	-2.010093759969E+00	-1.158694226407E+00	9.546252120016E-01
12	9	F	2.010093759969E+00	1.158694226407E+00	-9.546252120016E-01
13	109	F	-8.207154604467E-01	-2.317288538156E+00	3.798872741481E+00
14	109	F	8.207154604467E-01	2.317288538156E+00	-3.798872741481E+00
15	1	H	-1.531320705997E+00	-2.993522574597E+00	3.557566505132E+00
16	1	H	1.531320705997E+00	2.993522574597E+00	-3.557566505132E+00

(111) surface, 25% coverage

1	9	F	-1.873271718305E+00	3.381959753790E+00	2.304357097233E+00
2	9	F	1.873271718305E+00	-3.381959753790E+00	-2.304357097233E+00
3	9	F	4.496626758716E-02	4.959086668690E-02	2.325072840907E+00
4	9	F	-4.496626758716E-02	-4.959086668690E-02	-2.325072840907E+00
5	9	F	1.489324737247E+00	3.121221438468E+00	2.676034512641E+00
6	9	F	-1.489324737247E+00	-3.121221438468E+00	-2.676034512641E+00
7	9	F	3.879071634032E+00	-7.129879534649E-03	2.324739139450E+00
8	9	F	-3.879071634032E+00	7.129879534649E-03	-2.324739139450E+00
9	220	CA	-1.947154223848E+00	1.118170772731E+00	1.662434905485E+00
10	220	CA	1.947154223848E+00	-1.118170772731E+00	-1.662434905485E+00
11	220	CA	1.614525252818E-01	-2.158952677323E+00	1.635193552382E+00
12	220	CA	-1.614525252818E-01	2.158952677323E+00	-1.635193552382E+00
13	220	CA	1.915342301166E+00	1.116729940839E+00	1.519309430652E+00
14	220	CA	-1.915342301166E+00	-1.116729940839E+00	-1.519309430652E+00
15	220	CA	3.869137734866E+00	-2.261038506821E+00	1.512497751215E+00

16	220	CA	-3.869137734866E+00	2.261038506821E+00	-1.512497751215E+00
17	9	F	-3.009605246447E-02	2.224429836187E+00	6.884592241849E-01
18	9	F	3.009605246447E-02	-2.224429836187E+00	-6.884592241849E-01
19	9	F	2.019187395335E+00	-1.075032043640E+00	6.677028754959E-01
20	9	F	-2.019187395335E+00	1.075032043640E+00	-6.677028754959E-01
21	9	F	-1.006997239251E-01	-4.407942603071E+00	8.772482950725E-01
22	9	F	1.006997239251E-01	4.407942603071E+00	-8.772482950725E-01
23	9	F	-1.916268106365E+00	-1.250515559211E+00	8.815709420667E-01
24	9	F	1.916268106365E+00	1.250515559211E+00	-8.815709420667E-01
25	109	F	-5.897842029139E-01	-2.683872046182E+00	3.827942351982E+00
26	109	F	5.897842029139E-01	2.683872046182E+00	-3.827942351982E+00
27	1	H	-1.446452628759E+00	-3.108987474275E+00	3.452169669481E+00
28	1	H	1.446452628759E+00	3.108987474275E+00	-3.452169669481E+00

(110) surface, 100% coverage

1	220	CA	9.735490505145E-01	1.372463855412E+00	4.691722744229E+00
2	220	CA	-9.735490505145E-01	-1.372463855412E+00	-4.691722744229E+00
3	9	F	-9.726509997281E-01	2.679157105085E+00	4.859659103540E+00
4	9	F	9.726509997281E-01	-2.679157105085E+00	-4.859659103540E+00
5	9	F	9.726787106401E-01	-5.785838112376E-02	-4.897179309244E+00
6	9	F	-9.726787106401E-01	5.785838112376E-02	4.897179309244E+00
7	220	CA	-9.724796706846E-01	-1.379327164926E+00	3.012826543947E+00
8	220	CA	9.724796706846E-01	1.379327164926E+00	-3.012826543947E+00
9	9	F	9.731808004583E-01	-1.068621726256E-02	2.901298208478E+00
10	9	F	-9.731808004583E-01	1.068621726256E-02	-2.901298208478E+00
11	9	F	-9.731828583123E-01	2.747848419321E+00	-2.892990146600E+00
12	9	F	9.731828583123E-01	-2.747848419321E+00	2.892990146600E+00
13	220	CA	9.734171426759E-01	1.375940655502E+00	9.263798947039E-01
14	220	CA	-9.734171426759E-01	-1.375940655502E+00	-9.263798947039E-01
15	9	F	-9.730141800895E-01	2.751129400086E+00	9.859452265408E-01
16	9	F	9.730141800895E-01	-2.751129400086E+00	-9.859452265408E-01
17	9	F	9.730148739033E-01	-1.269710934598E-03	-9.887147329187E-01
18	9	F	-9.730148739033E-01	1.269710934598E-03	9.887147329187E-01
19	109	F	9.694375810767E-01	1.654061806104E+00	7.259856570428E+00
20	109	F	-9.694375810767E-01	-1.654061806104E+00	-7.259856570428E+00
21	1	H	9.696185066527E-01	1.192756678241E+00	8.076847067926E+00
22	1	H	-9.696185066527E-01	-1.192756678241E+00	-8.076847067926E+00

(110) surface, 50% coverage

1	220	CA	1.868168098976E+00	2.628860947204E+00	4.719206996255E+00
2	220	CA	-2.023659035898E+00	2.633550856576E+00	4.668460780087E+00
3	220	CA	-7.707401761300E-02	-1.173357884546E-01	-4.707255341112E+00
4	220	CA	3.814368551590E+00	-1.189167721721E-01	-4.660140765075E+00
5	9	F	-9.519750137734E-02	-1.565605733962E+00	4.874193939177E+00
6	9	F	3.830006933961E+00	-1.565339243394E+00	4.882453087347E+00
7	9	F	1.877464801614E+00	1.188779260362E+00	-4.885670478810E+00
8	9	F	-2.037998230878E+00	1.189754600473E+00	-4.879820517286E+00
9	9	F	1.887708262352E+00	-1.422067226255E+00	-4.867815290195E+00
10	9	F	-2.045620531509E+00	-1.421380320963E+00	-4.851308580445E+00
11	9	F	-1.010800631706E-01	1.322983877766E+00	4.877268555520E+00

12	9	F	3.831560310832E+00	1.324131160404E+00	4.892582184157E+00
13	220	CA	-7.757278602693E-02	-1.207643299790E-01	3.017426475194E+00
14	220	CA	3.810604343845E+00	-1.198669362198E-01	3.024030745120E+00
15	220	CA	1.864937161406E+00	2.634271042575E+00	-3.012087034156E+00
16	220	CA	-2.023029613507E+00	2.633936908718E+00	-3.010610064270E+00
17	9	F	1.869528163962E+00	1.248702739671E+00	2.917673745016E+00
18	9	F	-2.029117205086E+00	1.248595099580E+00	2.888039695403E+00
19	9	F	-7.586141140673E-02	-1.499164425964E+00	-2.903565561486E+00
20	9	F	3.809559982519E+00	-1.502247391074E+00	-2.876103890503E+00
21	9	F	-7.900475328907E-02	1.263718798223E+00	-2.906801372535E+00
22	9	F	3.812049750422E+00	1.266584811813E+00	-2.883741973229E+00
23	9	F	1.868081889957E+00	-1.491425983376E+00	2.914407350074E+00
24	9	F	-2.025962211581E+00	-1.486932711307E+00	2.888692496179E+00
25	220	CA	1.866080386113E+00	2.632711335653E+00	9.352323119676E-01
26	220	CA	-2.024950270219E+00	2.632330821341E+00	9.247067508387E-01
27	220	CA	-7.803811957940E-02	-1.191287006818E-01	-9.252600617087E-01
28	220	CA	3.810527879802E+00	-1.200176162523E-01	-9.150829354678E-01
29	9	F	-7.688088020963E-02	-1.496886801156E+00	9.914629784166E-01
30	9	F	3.810219091926E+00	-1.496242298305E+00	9.930810986228E-01
31	9	F	1.862496956958E+00	1.256530851777E+00	-9.842461299173E-01
32	9	F	-2.021735157880E+00	1.253985495113E+00	-9.830235893005E-01
33	9	F	1.862855943274E+00	-1.494040360328E+00	-9.801034351711E-01
34	9	F	-2.021648112340E+00	-1.494173199199E+00	-9.836573537735E-01
35	9	F	-7.516619294117E-02	1.257360073742E+00	9.939628768886E-01
36	9	F	3.807601018276E+00	1.258859583719E+00	9.916726920246E-01
37	109	F	1.707614952328E+00	2.672748104271E+00	7.251904542102E+00
38	1	H	1.710888716617E+00	2.201334536519E+00	8.066477738167E+00
39	109	F	-2.850955413314E-01	-5.261953888698E-01	-7.339529567333E+00
40	1	H	-2.803900480376E-01	-5.999967758788E-02	-8.157113096784E+00

(110) surface, 25% coverage

1	220	CA	1.904991178929E+00	2.692588215650E+00	4.723712169654E+00
2	220	CA	1.902205175726E+00	-2.816933048463E+00	4.679296002876E+00
3	220	CA	-1.987948154055E+00	2.693058102960E+00	4.671130847023E+00
4	220	CA	-1.989170785039E+00	-2.815812798506E+00	4.680500887502E+00
5	220	CA	-4.156164340169E-02	-6.561635012633E-02	-4.719103932795E+00
6	220	CA	-4.326483061395E-02	5.449867384401E+00	-4.675939708122E+00
7	220	CA	3.849984523384E+00	-6.482839215888E-02	-4.669469033552E+00
8	220	CA	3.848870130172E+00	5.447091588067E+00	-4.675819079003E+00
9	9	F	-6.136907202490E-02	3.995953903827E+00	4.858874712027E+00
10	9	F	-4.031853387685E-02	-1.513914174357E+00	4.889178769186E+00
11	9	F	3.867865452040E+00	3.997630330186E+00	4.868289146568E+00
12	9	F	3.845635944711E+00	-1.513211946511E+00	4.889655246269E+00
13	9	F	1.912875245559E+00	1.246844208492E+00	-4.888846542447E+00
14	9	F	1.903206821563E+00	-4.262626109299E+00	-4.872256454209E+00
15	9	F	-2.001467894262E+00	1.247067395801E+00	-4.886012358991E+00
16	9	F	-1.989313910704E+00	-4.263548881823E+00	-4.870878608343E+00
17	9	F	1.927440796232E+00	-1.365373962731E+00	-4.856813073183E+00
18	9	F	1.900621576305E+00	4.143976364079E+00	-4.884048900536E+00
19	9	F	-2.012929582297E+00	-1.365616084886E+00	-4.840339426565E+00
20	9	F	-1.986906773686E+00	4.144019682790E+00	-4.886203500071E+00

21	9	F	-6.504771705583E-02	1.379698969508E+00	4.871526005853E+00
22	9	F	-4.397565271619E-02	-4.114965164233E+00	4.879303410347E+00
23	9	F	3.869530396650E+00	1.381822704192E+00	4.881068598908E+00
24	9	F	3.848550764605E+00	-4.114712579835E+00	4.879338706775E+00
25	220	CA	-4.185727987051E-02	-5.742377157644E-02	3.017789160277E+00
26	220	CA	-4.298165921502E-02	5.438147873560E+00	3.017310364245E+00
27	220	CA	3.848420838792E+00	-5.734805344550E-02	3.023046822567E+00
28	220	CA	3.846751748945E+00	5.438677298054E+00	3.017016610434E+00
29	220	CA	1.900568738821E+00	2.687943099774E+00	-3.021452034524E+00
30	220	CA	1.903081591491E+00	-2.808289740636E+00	-3.010307953364E+00
31	220	CA	-1.987587973493E+00	2.688158992894E+00	-3.024019140948E+00
32	220	CA	-1.988484733732E+00	-2.809028247148E+00	-3.005552388068E+00
33	9	F	1.904820807834E+00	1.308568350052E+00	2.916140762026E+00
34	9	F	1.902193197356E+00	-4.196664123398E+00	2.897595548303E+00
35	9	F	-1.991242919183E+00	1.307212565132E+00	2.884249681599E+00
36	9	F	-1.989792164053E+00	-4.195831680039E+00	2.898134035239E+00
37	9	F	-4.374606884517E-02	4.060857175736E+00	-2.898290612376E+00
38	9	F	-3.960046040579E-02	-1.441269550768E+00	-2.904343088192E+00
39	9	F	3.848697520675E+00	4.059366134672E+00	-2.901307619697E+00
40	9	F	3.845817716570E+00	-1.443068598301E+00	-2.872306636035E+00
41	9	F	-4.375408983421E-02	1.320816148221E+00	-2.915367714476E+00
42	9	F	-4.304393894841E-02	-4.181030475369E+00	-2.890365382497E+00
43	9	F	3.848476057429E+00	1.324397996055E+00	-2.890758237904E+00
44	9	F	3.848840853892E+00	-4.181986541758E+00	-2.891721807526E+00
45	9	F	1.902953894540E+00	-1.430942337098E+00	2.900896257832E+00
46	9	F	1.903995016531E+00	4.072055127551E+00	2.908862974619E+00
47	9	F	-1.989146905950E+00	-1.429744237706E+00	2.905035945957E+00
48	9	F	-1.990392172927E+00	4.074948319208E+00	2.880978657361E+00
49	220	CA	1.902739254961E+00	2.690267338581E+00	9.324006353900E-01
50	220	CA	1.901595597577E+00	-2.812425034227E+00	9.260604365350E-01
51	220	CA	-1.989062471655E+00	2.690688021388E+00	9.207667386189E-01
52	220	CA	-1.988685943264E+00	-2.813155702662E+00	9.266598019269E-01
53	220	CA	-4.251004525592E-02	-5.963013012689E-02	-9.302911102857E-01
54	220	CA	-4.247178087633E-02	5.441762440934E+00	-9.211861922921E-01
55	220	CA	3.847749523114E+00	-5.967224440746E-02	-9.189841517632E-01
56	220	CA	3.847235928634E+00	5.440950933446E+00	-9.211323538444E-01
57	9	F	-3.987167750928E-02	4.064692441719E+00	9.863713073762E-01
58	9	F	-4.461010210277E-02	-1.437489554626E+00	9.922577706997E-01
59	9	F	3.845169231081E+00	4.065400176923E+00	9.863112235755E-01
60	9	F	3.850014026495E+00	-1.437758253666E+00	9.931121618912E-01
61	9	F	1.899114923419E+00	1.315704322201E+00	-9.895964775416E-01
62	9	F	1.902525424199E+00	-4.190874464276E+00	-9.841377142624E-01
63	9	F	-1.985755714247E+00	1.313852134255E+00	-9.900129064086E-01
64	9	F	-1.989514087292E+00	-4.191912848361E+00	-9.819830002845E-01
65	9	F	1.898452836487E+00	-1.434506741960E+00	-9.792595484759E-01
66	9	F	1.902771257772E+00	4.067273697372E+00	-9.895202693647E-01
67	9	F	-1.984686687178E+00	-1.434964769611E+00	-9.822189077310E-01
68	9	F	-1.989890142945E+00	4.067494439439E+00	-9.905370497122E-01
69	9	F	-3.892563377920E-02	1.315680318176E+00	9.896711756585E-01
70	9	F	-4.285849924881E-02	-4.187784996000E+00	9.899086849931E-01
71	9	F	3.844104342622E+00	1.316703423105E+00	9.887066022454E-01
72	9	F	3.847612106974E+00	-4.187297545209E+00	9.897651248247E-01

73	109	F	1.774023769064E+00	2.729956867361E+00	7.256225908960E+00
74	1	H	1.776122588945E+00	2.266129908405E+00	8.075405437804E+00
75	109	F	-2.208000936005E-01	-5.325107191542E-01	-7.320078074383E+00
76	1	H	-2.165959792483E-01	-7.355453970844E-02	-8.142093344171E+00

(100) surface, 100% coverage

1	9	F	-9.194857957268E-02	1.126890121001E+00	9.323782460780E+00
2	9	F	-9.194857957268E-02	1.126890121001E+00	-9.323782460780E+00
3	220	CA	-2.066178277749E+00	1.646283735892E+00	8.271500187187E+00
4	220	CA	-2.066178277749E+00	1.646283735892E+00	-8.271500187187E+00
5	9	F	-1.149777469387E-01	1.716461170971E+00	6.759250533844E+00
6	9	F	-1.149777469387E-01	1.716461170971E+00	-6.759250533844E+00
7	9	F	1.830686803769E+00	-2.767624588512E-01	7.041342482706E+00
8	9	F	1.830686803769E+00	-2.767624588512E-01	-7.041342482706E+00
9	220	CA	-1.197078430626E-01	-2.377280741046E-01	5.513631494223E+00
10	220	CA	-1.197078430626E-01	-2.377280741046E-01	-5.513631494223E+00
11	9	F	1.827288161891E+00	-2.312265177144E-01	4.197026205207E+00
12	9	F	1.827288161891E+00	-2.312265177144E-01	-4.197026205207E+00
13	9	F	-1.187579113753E-01	1.714525199055E+00	4.071939211503E+00
14	9	F	-1.187579113753E-01	1.714525199055E+00	-4.071939211503E+00
15	220	CA	1.828514972819E+00	1.715485814622E+00	2.754131967766E+00
16	220	CA	1.828514972819E+00	1.715485814622E+00	-2.754131967766E+00
17	9	F	-1.165838836517E-01	1.715191214603E+00	1.359682863359E+00
18	9	F	-1.165838836517E-01	1.715191214603E+00	-1.359682863359E+00
19	9	F	1.829230651667E+00	-2.307107842169E-01	1.394822038544E+00
20	9	F	1.829230651667E+00	-2.307107842169E-01	-1.394822038544E+00
21	220	CA	-1.161566640852E-01	-2.313154858805E-01	0.000000000000E+00
22	109	F	-1.777512036982E+00	-6.206081450174E-01	1.009033689242E+01
23	109	F	-1.777512036982E+00	-6.206081450174E-01	-1.009033689242E+01
24	1	H	-9.179338405953E-01	-1.502278096491E-01	1.009510869026E+01
25	1	H	-9.179338405953E-01	-1.502278096491E-01	-1.009510869026E+01

(100) surface, 50% coverage

1	9	F	1.466611705386E-01	1.467676319396E+00	9.414898241809E+00
2	9	F	3.811725355920E+00	1.852353811726E+00	9.342086125234E+00
3	9	F	1.466611705386E-01	1.467676319396E+00	-9.414898241809E+00
4	9	F	3.811725355920E+00	1.852353811726E+00	-9.342086125234E+00
5	220	CA	-2.010350237742E+00	1.914072166641E+00	8.351790682410E+00
6	220	CA	1.973894815571E+00	1.909872326421E+00	8.215139205865E+00
7	220	CA	1.973894815571E+00	1.909872326421E+00	-8.215139205865E+00
8	220	CA	-2.010350237742E+00	1.914072166641E+00	-8.351790682410E+00
9	9	F	-2.811068675149E-02	1.957177187916E+00	6.780482406509E+00
10	9	F	3.874600060783E+00	1.925487871535E+00	6.696011015899E+00
11	9	F	-2.811068675149E-02	1.957177187916E+00	-6.780482406509E+00
12	9	F	3.874600060783E+00	1.925487871535E+00	-6.696011015899E+00
13	9	F	-1.985472203022E+00	-2.289366593966E-02	7.080655697003E+00
14	9	F	1.940014704322E+00	-2.638009020117E-02	7.077804223582E+00
15	9	F	1.940014704322E+00	-2.638009020117E-02	-7.077804223582E+00
16	9	F	-1.985472203022E+00	-2.289366593966E-02	-7.080655697003E+00
17	220	CA	-3.888834605959E-02	1.231641730653E-03	5.535965151468E+00

18	220	CA	3.889763239475E+00	-1.968992106257E-02	5.490192959016E+00
19	220	CA	-3.888834605959E-02	1.231641730654E-03	-5.535965151468E+00
20	220	CA	3.889763239475E+00	-1.968992106257E-02	-5.490192959016E+00
21	9	F	-1.958586457605E+00	-7.003399946217E-03	4.197939701955E+00
22	9	F	1.919301499129E+00	-7.003044942217E-03	4.218279044914E+00
23	9	F	1.919301499129E+00	-7.003044942216E-03	-4.218279044914E+00
24	9	F	-1.958586457605E+00	-7.003399946216E-03	-4.197939701955E+00
25	9	F	-2.413629306563E-02	1.940662989913E+00	4.083468123838E+00
26	9	F	3.876340946343E+00	1.936171554504E+00	4.041202681592E+00
27	9	F	-2.413629306563E-02	1.940662989913E+00	-4.083468123838E+00
28	9	F	3.876340946343E+00	1.936171554504E+00	-4.041202681592E+00
29	220	CA	-1.960329219409E+00	1.939109411018E+00	2.746444497604E+00
30	220	CA	1.920996933967E+00	1.938651274496E+00	2.761381673993E+00
31	220	CA	1.920996933967E+00	1.938651274496E+00	-2.761381673993E+00
32	220	CA	-1.960329219409E+00	1.939109411018E+00	-2.746444497604E+00
33	9	F	-1.750901433957E-02	1.938259216151E+00	1.359042323016E+00
34	9	F	3.871315149505E+00	1.938519612745E+00	1.352989848611E+00
35	9	F	-1.750901433957E-02	1.938259216151E+00	-1.359042323016E+00
36	9	F	3.871315149505E+00	1.938519612745E+00	-1.352989848611E+00
37	9	F	-1.962511132480E+00	-7.250723656567E-03	1.394645364338E+00
38	9	F	1.924000969478E+00	-7.482853700916E-03	1.402216013666E+00
39	9	F	1.924000969478E+00	-7.482853700916E-03	-1.402216013666E+00
40	9	F	-1.962511132480E+00	-7.250723656567E-03	-1.394645364338E+00
41	220	CA	-1.562129433459E-02	-7.381564506718E-03	2.426260859039E-51
42	220	CA	3.870058308705E+00	-8.146741120792E-03	2.426260859039E-51
43	109	F	-1.625851470318E+00	-2.177516349320E-01	1.001071085020E+01
44	1	H	-7.539976889285E-01	2.446324902364E-01	1.001444221871E+01
45	109	F	-1.625851470318E+00	-2.177516349320E-01	-1.001071085020E+01
46	1	H	-7.539976889285E-01	2.446324902364E-01	-1.001444221871E+01

(100) surface, 25% coverage

1	9	F	9.720288238013E-02	-2.383583154628E+00	9.327491376968E+00
2	9	F	3.504141920893E-02	1.672244161700E+00	9.438950736618E+00
3	9	F	3.896253903706E+00	-2.114462996874E+00	9.343769694216E+00
4	9	F	3.829616839306E+00	2.030549917822E+00	9.349073986105E+00
5	9	F	9.720288238012E-02	-2.383583154628E+00	-9.327491376968E+00
6	9	F	3.504141920893E-02	1.672244161700E+00	-9.438950736618E+00
7	9	F	3.896253903706E+00	-2.114462996874E+00	-9.343769694216E+00
8	9	F	3.829616839306E+00	2.030549917822E+00	-9.349073986105E+00
9	220	CA	-1.896498221140E+00	-1.948319221178E+00	8.382853148876E+00
10	220	CA	-2.037003167800E+00	1.903418119356E+00	8.274841906832E+00
11	220	CA	1.993484649506E+00	-2.006914097733E+00	8.262778130171E+00
12	220	CA	1.941094894884E+00	1.949333378963E+00	8.263655815976E+00
13	220	CA	1.993484649506E+00	-2.006914097733E+00	-8.262778130171E+00
14	220	CA	1.941094894884E+00	1.949333378963E+00	-8.263655815976E+00
15	220	CA	-1.896498221140E+00	-1.948319221178E+00	-8.382853148876E+00
16	220	CA	-2.037003167800E+00	1.903418119356E+00	-8.274841906832E+00
17	9	F	8.294216473661E-03	-1.925233169128E+00	6.717586097557E+00
18	9	F	-3.130201780992E-02	1.947465832777E+00	6.767406381606E+00
19	9	F	3.854022349075E+00	-1.956969697854E+00	6.688507541947E+00
20	9	F	3.895181491674E+00	1.921432618862E+00	6.703537020557E+00

21	9 F	8.294216473660E-03	-1.925233169128E+00	-6.717586097557E+00
22	9 F	-3.130201780992E-02	1.947465832777E+00	-6.767406381606E+00
23	9 F	3.854022349075E+00	-1.956969697854E+00	-6.688507541947E+00
24	9 F	3.895181491674E+00	1.921432618862E+00	-6.703537020557E+00
25	9 F	-1.971563347074E+00	-2.883608260150E-02	7.070038885366E+00
26	9 F	-1.959613593804E+00	3.884338241917E+00	7.164878152380E+00
27	9 F	1.947168098796E+00	-7.204215420688E-03	7.176008032206E+00
28	9 F	1.943401219719E+00	3.856206036394E+00	7.074193188402E+00
29	9 F	1.947168098796E+00	-7.204215420688E-03	-7.176008032206E+00
30	9 F	1.943401219719E+00	3.856206036394E+00	-7.074193188402E+00
31	9 F	-1.971563347074E+00	-2.883608260150E-02	-7.070038885366E+00
32	9 F	-1.959613593804E+00	3.884338241917E+00	-7.164878152380E+00
33	220 CA	-2.130885365670E-02	1.142600697591E-03	5.521221916218E+00
34	220 CA	-1.410576297572E-02	3.899502243540E+00	5.533636514206E+00
35	220 CA	3.893922472144E+00	-2.644880219981E-02	5.498772591742E+00
36	220 CA	3.890286709442E+00	3.887207607833E+00	5.508176471257E+00
37	220 CA	-2.130885365670E-02	1.142600697592E-03	-5.521221916218E+00
38	220 CA	-1.410576297572E-02	3.899502243540E+00	-5.533636514206E+00
39	220 CA	3.893922472144E+00	-2.644880219981E-02	-5.498772591742E+00
40	220 CA	3.890286709442E+00	3.887207607833E+00	-5.508176471257E+00
41	9 F	-1.951172263584E+00	-1.600915239263E-03	4.199492874880E+00
42	9 F	-1.950257793892E+00	3.887525966755E+00	4.230241625539E+00
43	9 F	1.932978632811E+00	-4.665145652781E-03	4.236898416242E+00
44	9 F	1.933996865217E+00	3.891454587371E+00	4.218306259008E+00
45	9 F	1.932978632811E+00	-4.665145652780E-03	-4.236898416242E+00
46	9 F	1.933996865217E+00	3.891454587371E+00	-4.218306259008E+00
47	9 F	-1.951172263584E+00	-1.600915239263E-03	-4.199492874880E+00
48	9 F	-1.950257793892E+00	3.887525966755E+00	-4.230241625539E+00
49	9 F	-1.261861413530E-02	-1.949154767818E+00	4.053111446290E+00
50	9 F	-8.520249925752E-03	1.946801903394E+00	4.075281260291E+00
51	9 F	3.888355201356E+00	-1.953495338396E+00	4.034518341083E+00
52	9 F	3.883796400229E+00	1.942273206216E+00	4.047077208780E+00
53	9 F	-1.261861413530E-02	-1.949154767818E+00	-4.053111446290E+00
54	9 F	-8.520249925752E-03	1.946801903394E+00	-4.075281260291E+00
55	9 F	3.888355201356E+00	-1.953495338396E+00	-4.034518341083E+00
56	9 F	3.883796400229E+00	1.942273206216E+00	-4.047077208780E+00
57	220 CA	-1.950738095630E+00	-1.948646168052E+00	2.746169540042E+00
58	220 CA	-1.953230322859E+00	1.943347680844E+00	2.754075897317E+00
59	220 CA	1.933805376664E+00	-1.951182090256E+00	2.754950490464E+00
60	220 CA	1.935279557496E+00	1.945476370290E+00	2.761684115434E+00
61	220 CA	1.933805376664E+00	-1.951182090256E+00	-2.754950490464E+00
62	220 CA	1.935279557496E+00	1.945476370290E+00	-2.761684115434E+00
63	220 CA	-1.950738095630E+00	-1.948646168052E+00	-2.746169540042E+00
64	220 CA	-1.953230322859E+00	1.943347680844E+00	-2.754075897317E+00
65	9 F	-7.019353467222E-03	-1.949229378076E+00	1.351611221138E+00
66	9 F	-7.694902067544E-03	1.943320387770E+00	1.359763469368E+00
67	9 F	3.881983426335E+00	-1.949114185528E+00	1.349694215504E+00
68	9 F	3.882494711047E+00	1.943617948038E+00	1.354197777746E+00
69	9 F	-7.019353467222E-03	-1.949229378076E+00	-1.351611221138E+00
70	9 F	-7.694902067544E-03	1.943320387770E+00	-1.359763469368E+00
71	9 F	3.881983426335E+00	-1.949114185528E+00	-1.349694215504E+00
72	9 F	3.882494711047E+00	1.943617948038E+00	-1.354197777746E+00

73	9	F	-1.952718994530E+00	-1.642722427215E-03	1.395394895883E+00
74	9	F	-1.952919045132E+00	3.887964644217E+00	1.401309288984E+00
75	9	F	1.936155080448E+00	-1.363929477809E-03	1.406477200011E+00
76	9	F	1.935804957625E+00	3.887615087942E+00	1.401126514871E+00
77	9	F	1.936155080448E+00	-1.363929477809E-03	-1.406477200011E+00
78	9	F	1.935804957625E+00	3.887615087942E+00	-1.401126514871E+00
79	9	F	-1.952718994530E+00	-1.642722427214E-03	-1.395394895883E+00
80	9	F	-1.952919045132E+00	3.887964644217E+00	-1.401309288984E+00
81	220	CA	-6.209940214104E-03	-1.287068520730E-03	3.074169222090E-50
82	220	CA	-6.908136979407E-03	3.887616132947E+00	3.074169222090E-50
83	220	CA	3.882285815084E+00	-2.315739169832E-03	3.074169222090E-50
84	220	CA	3.881609583230E+00	3.887989593421E+00	3.074169222090E-50
85	109	F	-1.658700276791E+00	-8.496073176892E-02	1.000576528210E+01
86	1	H	-7.923980572569E-01	3.911923664562E-01	1.001547427735E+01
87	109	F	-1.658700276791E+00	-8.496073176892E-02	-1.000576528210E+01
88	1	H	-7.923980572569E-01	3.911923664562E-01	-1.001547427735E+01

2. SrF2:

(111) surface, 100% coverage

1	9	F	-1.033363115623E+00	1.833650483242E+00	2.475433147356E+00
2	9	F	-1.033156452401E+00	1.745666403398E+00	-2.475433147356E+00
3	238	SR	-1.034453951997E+00	-6.142232169736E-01	1.690581471530E+00
4	238	SR	1.034453951996E+00	6.142232169746E-01	-1.690581471530E+00
5	9	F	1.033362132472E+00	5.998749233479E-01	8.254826511571E-01
6	9	F	-1.033362132471E+00	-5.998749233490E-01	-8.254826511571E-01
7	109	F	-1.025107609167E+00	1.284868673780E-01	4.195398325048E+00
8	1	H	-1.028565668754E+00	1.025287458947E+00	3.784604538686E+00
9	109	F	1.025107609167E+00	-1.284868673780E-01	-4.195398325048E+00
10	1	H	1.028565668754E+00	-1.025287458947E+00	-3.784604538686E+00

(111) surface, 50% coverage

1	9	F	1.129497003163E-01	-2.936491029848E-02	2.571343478637E+00
2	9	F	4.104861152949E+00	-6.528096961124E-03	2.371947858710E+00
3	9	F	-1.129497003196E-01	2.936491029745E-02	-2.571343478637E+00
4	9	F	4.161217119131E+00	6.528096960095E-03	-2.371947858710E+00
5	238	SR	-2.113172541412E+00	1.196976272550E+00	1.686556573533E+00
6	238	SR	2.094452557192E+00	1.204902091384E+00	1.676590706341E+00
7	238	SR	2.113172541410E+00	-1.196976272549E+00	-1.686556573533E+00
8	238	SR	-2.094452557193E+00	-1.204902091383E+00	-1.676590706341E+00
9	9	F	2.046489169736E+00	-1.231620375690E+00	8.262746182015E-01
10	9	F	-2.038861949057E+00	-1.159175554748E+00	8.117033467591E-01
11	9	F	-2.046489169735E+00	1.231620375689E+00	-8.262746182015E-01
12	9	F	2.038861949058E+00	1.159175554747E+00	-8.117033467591E-01
13	109	F	-1.461233543818E+00	7.440034009931E-01	4.189846620105E+00
14	1	H	-6.704500890947E-01	3.367035866896E-01	3.731656014586E+00
15	109	F	1.461233543818E+00	-7.440034009931E-01	-4.189846620105E+00

16	1	H	6.704500890947E-01	-3.367035866896E-01	-3.731656014586E+00

(111) surface, 25% coverage

1	9	F	2.778071642621E-01	-1.598148947422E-01	2.712548327994E+00
2	9	F	-2.094256511543E+00	3.619705320773E+00	2.424672280726E+00
3	9	F	4.083435727702E+00	3.655233855473E-03	2.423283132556E+00
4	9	F	2.088591978363E+00	3.567067694687E+00	2.428715395665E+00
5	9	F	-2.038782624501E+00	3.538928452507E+00	-2.424672280726E+00
6	9	F	-2.778071642655E-01	1.598148947412E-01	-2.712548327994E+00
7	9	F	2.044447157677E+00	3.591566078593E+00	-2.428715395665E+00
8	9	F	4.182642544378E+00	-3.655233856501E-03	-2.423283132556E+00
9	238	SR	-2.165755480867E+00	1.250142304211E+00	1.719860129789E+00
10	238	SR	1.673850506991E-02	-2.413788112407E+00	1.625534065949E+00
11	238	SR	2.098634774100E+00	1.193057330106E+00	1.625023530460E+00
12	238	SR	4.134213419032E+00	-2.387106238378E+00	1.742469101370E+00
13	238	SR	-1.673850507103E-02	2.413788112408E+00	-1.625534065949E+00
14	238	SR	2.165755480865E+00	-1.250142304210E+00	-1.719860129789E+00
15	238	SR	-4.134213419033E+00	2.387106238379E+00	-1.742469101370E+00
16	238	SR	-2.098634774101E+00	-1.193057330105E+00	-1.625023530460E+00
17	9	F	-1.487032249573E-02	2.309318951582E+00	8.791687027146E-01
18	9	F	2.067862288630E+00	-1.193826202602E+00	7.682793801851E-01
19	9	F	-4.160355291012E+00	2.402387152236E+00	7.392995184113E-01
20	9	F	-2.007075169287E+00	-1.142063024584E+00	8.789518946167E-01
21	9	F	-2.067862288629E+00	1.193826202601E+00	-7.682793801851E-01
22	9	F	1.487032249684E-02	-2.309318951583E+00	-8.791687027146E-01
23	9	F	2.007075169288E+00	1.142063024583E+00	-8.789518946167E-01
24	9	F	4.160355291013E+00	-2.402387152237E+00	-7.392995184113E-01
25	109	F	-1.362971176414E+00	7.803585329229E-01	4.125521022111E+00
26	1	H	-5.678497265338E-01	3.246983945571E-01	3.674797064383E+00
27	109	F	1.362971176414E+00	-7.803585329229E-01	-4.125521022111E+00
28	1	H	5.678497265338E-01	-3.246983945571E-01	-3.674797064383E+00

(110) surface, 100% coverage

1	238	SR	1.033259784010E+00	1.461250000000E+00	5.003948972420E+00
2	238	SR	-1.033259784010E+00	-1.461250000000E+00	-5.003948972420E+00
3	9	F	-1.033259784010E+00	2.838512608797E+00	5.150386023294E+00
4	9	F	1.033259784010E+00	-8.398739120270E-02	-5.150386023294E+00
5	9	F	1.033259784010E+00	-2.838512608797E+00	-5.150386023294E+00
6	9	F	-1.033259784010E+00	8.398739120270E-02	5.150386023294E+00
7	238	SR	-1.033259784010E+00	-1.461250000000E+00	3.178706935499E+00
8	238	SR	1.033259784010E+00	1.461250000000E+00	-3.178706935499E+00
9	9	F	1.033259784010E+00	-2.063629581335E-03	3.077019974441E+00
10	9	F	-1.033259784010E+00	2.920436370419E+00	-3.077019974441E+00
11	9	F	-1.033259784010E+00	2.063629581335E-03	-3.077019974441E+00
12	9	F	1.033259784010E+00	-2.920436370419E+00	3.077019974441E+00
13	238	SR	1.033259784010E+00	1.461250000000E+00	9.936767255593E-01
14	238	SR	-1.033259784010E+00	-1.461250000000E+00	-9.936767255593E-01
15	9	F	-1.033259784010E+00	2.922331378381E+00	1.048494731519E+00

16	9	F	1.033259784010E+00	-1.686216190769E-04	-1.048494731519E+00
17	9	F	1.033259784010E+00	-2.922331378381E+00	-1.048494731519E+00
18	9	F	-1.033259784010E+00	1.686216190769E-04	1.048494731519E+00
19	109	F	1.033259780000E+00	1.461250000000E+00	7.771224162931E+00
20	109	F	-1.033259780000E+00	4.383750000000E+00	-7.771224162931E+00
21	1	H	1.033259780000E+00	1.461250000000E+00	8.711071486663E+00
22	1	H	-1.033259780000E+00	4.383750000000E+00	-8.711071486663E+00

(110) surface, 50% coverage

1	238	SR	2.177365392789E+00	2.654239455628E+00	5.086363348522E+00
2	238	SR	-2.177365392789E+00	2.654239455628E+00	5.086363348522E+00
3	238	SR	-1.111483713991E-15	-1.608961857570E-01	-5.005677640158E+00
4	238	SR	4.133039136040E+00	-1.618378215110E-01	-5.002846317691E+00
5	9	F	1.215039912491E-15	-1.281517686665E+00	5.472053297136E+00
6	9	F	4.133039136040E+00	-1.855608689962E+00	4.881940914046E+00
7	9	F	2.068619693836E+00	1.208241369102E+00	-5.145038264131E+00
8	9	F	-2.068619693836E+00	1.208241369102E+00	-5.145038264131E+00
9	9	F	2.065151074672E+00	-1.530857413093E+00	-5.144740510352E+00
10	9	F	-2.065151074672E+00	-1.530857413093E+00	-5.144740510352E+00
11	9	F	1.106069855864E-15	1.600816933164E+00	4.981295790714E+00
12	9	F	4.133039136040E+00	1.253273308939E+00	5.147965958460E+00
13	238	SR	6.932085857215E-16	-1.118723196690E-01	3.121933928346E+00
14	238	SR	4.133039136040E+00	-2.135463862520E-01	3.139956414392E+00
15	238	SR	2.067353740739E+00	2.760886165569E+00	-3.176316926324E+00
16	238	SR	-2.067353740739E+00	2.760886165569E+00	-3.176316926324E+00
17	9	F	2.110458331824E+00	1.247253092730E+00	3.055119086165E+00
18	9	F	-2.110458331824E+00	1.247253092730E+00	3.055119086165E+00
19	9	F	-6.833961708602E-16	-1.622333909348E+00	-3.077742740433E+00
20	9	F	4.133039136040E+00	-1.624978684572E+00	-3.081668660393E+00
21	9	F	-6.851473910669E-16	1.303615218881E+00	-3.085629535103E+00
22	9	F	4.133039136040E+00	1.299058711272E+00	-3.075202838158E+00
23	9	F	1.941145465895E+00	-1.682404049025E+00	3.139216524109E+00
24	9	F	-1.941145465895E+00	-1.682404049025E+00	3.139216524109E+00
25	238	SR	2.060762610391E+00	2.758918858293E+00	1.012571020597E+00
26	238	SR	-2.060762610391E+00	2.758918858293E+00	1.012571020597E+00
27	238	SR	-2.239131519676E-16	-1.605923590050E-01	-1.008415187765E+00
28	238	SR	4.133039136040E+00	-1.607969575940E-01	-9.980912959220E-01
29	9	F	2.261666014034E-16	-1.601820150053E+00	1.018563821804E+00
30	9	F	4.133039136040E+00	-1.634737346127E+00	1.073028924942E+00
31	9	F	2.057582423198E+00	1.299584439404E+00	-1.047601176365E+00
32	9	F	-2.057582423198E+00	1.299584439404E+00	-1.047601176365E+00
33	9	F	2.075905304989E+00	-1.623495547648E+00	-1.043106851587E+00
34	9	F	-2.075905304989E+00	-1.623495547648E+00	-1.043106851587E+00
35	9	F	2.326627906883E-16	1.310292003867E+00	1.047820057447E+00
36	9	F	4.133039136040E+00	1.299524446285E+00	1.016671214089E+00
37	109	F	1.817729731588E+00	-1.506755523672E+00	7.023717373519E+00
38	109	F	-1.817729731588E+00	-1.506755523672E+00	7.023717373519E+00
39	1	H	-1.021479936875E+00	-1.238098235330E+00	6.479616111871E+00
40	1	H	1.021479936875E+00	-1.238098235330E+00	6.479616111871E+00

(110) surface, 25% coverage

1	238	SR	2.067058827348E+00	2.922046949538E+00	5.030646307023E+00
2	238	SR	-2.066893436378E+00	-2.922195944289E+00	4.991145405603E+00
3	238	SR	2.066240332680E+00	-2.922177645841E+00	4.992283868987E+00
4	238	SR	-2.066184965441E+00	2.922265325980E+00	4.990316752474E+00
5	9	F	2.182504189176E-04	-1.554141429379E+00	5.149892247199E+00
6	9	F	-1.273923181296E-02	1.547563086732E+00	5.141207071980E+00
7	9	F	-1.269561032197E-02	4.297138660940E+00	5.143201053923E+00
8	9	F	7.462257774853E-05	-4.290413296559E+00	5.151571935282E+00
9	9	F	4.132414631394E+00	-1.554156045354E+00	5.150508935548E+00
10	9	F	4.146410770151E+00	1.547231846687E+00	5.142129737972E+00
11	9	F	4.146143853780E+00	4.297124392119E+00	5.142043949268E+00
12	9	F	4.132441988487E+00	-4.290559102674E+00	5.150819087832E+00
13	238	SR	1.273731460994E-03	3.565587962888E-03	3.177616679820E+00
14	238	SR	9.397547017157E-04	5.841525953691E+00	3.180741751249E+00
15	238	SR	4.131960857679E+00	3.227134190278E-03	3.178760802683E+00
16	238	SR	4.131348268383E+00	5.841713842801E+00	3.179168132762E+00
17	9	F	2.066937019492E+00	1.460857360267E+00	3.087380199294E+00
18	9	F	-2.066398646471E+00	-1.459023594148E+00	3.075595953917E+00
19	9	F	2.066508276475E+00	-1.459664215100E+00	3.075133933236E+00
20	9	F	-2.066532038862E+00	1.458533228006E+00	3.067706324129E+00
21	9	F	2.066163924890E+00	-4.385300688146E+00	3.075911449210E+00
22	9	F	-2.066413178252E+00	4.386669024687E+00	3.068416646981E+00
23	9	F	2.066292574938E+00	4.383893350144E+00	3.087899155481E+00
24	9	F	-2.066588008008E+00	-4.385718415014E+00	3.076064524441E+00
25	238	SR	2.066752583391E+00	2.921887660857E+00	9.997046391569E-01
26	238	SR	-2.065748296747E+00	-2.921940102885E+00	9.896034520559E-01
27	238	SR	2.065866628380E+00	-2.921983056538E+00	9.907123139525E-01
28	238	SR	-2.066175582663E+00	2.922119843176E+00	9.916645692466E-01
29	9	F	-1.719693509669E-05	-1.461397084077E+00	1.046563128595E+00
30	9	F	2.095211051669E-03	1.461315726140E+00	1.047143805439E+00
31	9	F	2.148231114365E-03	4.383486061484E+00	1.049793299267E+00
32	9	F	3.000099856660E-04	-4.383227974623E+00	1.049374602513E+00
33	9	F	4.132944352822E+00	-1.462159638218E+00	1.048374368317E+00
34	9	F	4.131275502046E+00	1.461510753040E+00	1.048835200068E+00
35	9	F	4.131350510311E+00	4.383451669120E+00	1.049333163037E+00
36	9	F	4.132738184473E+00	-4.382956115235E+00	1.048704916820E+00
37	238	SR	-1.576507703459E-04	8.344038842445E-04	-1.000164218493E+00
38	238	SR	8.244597795109E-04	5.844284435163E+00	-9.899681539110E-01
39	238	SR	4.132696506565E+00	4.005756126860E-04	-9.913206188134E-01
40	238	SR	4.132791284819E+00	5.844538054123E+00	-9.907993046935E-01
41	9	F	2.064451272695E+00	1.461227244728E+00	-1.047478770885E+00
42	9	F	-2.064540632690E+00	-1.460773145190E+00	-1.049891863423E+00
43	9	F	2.064181150054E+00	-1.460993510575E+00	-1.049397517352E+00
44	9	F	-2.064674757845E+00	1.461097550263E+00	-1.048728623125E+00
45	9	F	2.066595309320E+00	-4.384337902649E+00	-1.049129148737E+00
46	9	F	-2.066282092807E+00	4.384702434405E+00	-1.048392938498E+00
47	9	F	2.066846057094E+00	4.384021802442E+00	-1.046670851214E+00
48	9	F	-2.066198252324E+00	-4.384544420823E+00	-1.048966255763E+00
49	238	SR	2.065444768231E+00	2.919048036085E+00	-3.177960854475E+00

50	238	SR	-2.065217771716E+00	-2.919202980198E+00	-3.180357419772E+00
51	238	SR	2.065045610013E+00	-2.918928902222E+00	-3.179220718936E+00
52	238	SR	-2.065449213152E+00	2.919120193036E+00	-3.178234138687E+00
53	9	F	3.349359360306E-04	4.382078227750E+00	-3.074623580755E+00
54	9	F	-7.496743102819E-05	-4.382120678695E+00	-3.075225704591E+00
55	9	F	3.062047432018E-05	-1.461322290184E+00	-3.088341924495E+00
56	9	F	-1.813921984487E-04	1.461655704505E+00	-3.087814516052E+00
57	9	F	4.132787316000E+00	4.381649111773E+00	-3.076074834181E+00
58	9	F	4.133243696973E+00	-4.381724034706E+00	-3.076667915732E+00
59	9	F	4.132881364605E+00	-1.464274925835E+00	-3.067893351265E+00
60	9	F	4.133133386192E+00	1.464024637287E+00	-3.067369992311E+00
61	238	SR	-9.070462835605E-05	5.315944869594E-04	-5.031230856600E+00
62	238	SR	3.797427591453E-04	5.844712808759E+00	-4.991655144966E+00
63	238	SR	4.132864023834E+00	1.252568599269E-04	-4.989927913539E+00
64	238	SR	4.133323591372E+00	5.844794771371E+00	-4.991674405729E+00
65	9	F	2.079701522768E+00	1.374824302334E+00	-5.141409786609E+00
66	9	F	-2.079572269198E+00	-1.374538210657E+00	-5.142516929850E+00
67	9	F	2.079492898503E+00	-1.374818251295E+00	-5.142410894884E+00
68	9	F	-2.079804435929E+00	1.375252359319E+00	-5.141728304599E+00
69	9	F	2.066140023278E+00	-4.477016696350E+00	-5.151107240106E+00
70	9	F	-2.065916416997E+00	4.476599347570E+00	-5.150275054864E+00
71	9	F	2.066117403904E+00	4.476472158576E+00	-5.150585998403E+00
72	9	F	-2.065802300325E+00	-4.476895889886E+00	-5.151769662383E+00
73	109	F	2.067092461087E+00	2.921433349186E+00	7.756948904022E+00
74	1	H	2.064000738537E+00	2.921184903219E+00	8.699163691211E+00
75	109	F	9.587256274143E-05	9.551118089181E-04	-7.757342295439E+00
76	1	H	-3.120074942259E-04	9.767407073637E-04	-8.699563877226E+00

(100) surface, 100% coverage

1	9	F	2.516058014189E-02	2.306683037906E+00	1.012206601313E+01
2	9	F	2.516058014189E-02	2.306683037906E+00	-1.012206601313E+01
3	238	SR	-1.931906488065E+00	1.771045387769E+00	8.768662296077E+00
4	238	SR	-1.931906488065E+00	1.771045387769E+00	-8.768662296077E+00
5	9	F	1.506605874085E-01	1.712713138510E+00	7.300433195570E+00
6	9	F	1.506605874085E-01	1.712713138510E+00	-7.300433195570E+00
7	9	F	-1.872854436523E+00	-3.155295666744E-01	7.304295197971E+00
8	9	F	-1.872854436523E+00	-3.155295666744E-01	-7.304295197971E+00
9	238	SR	1.839196975150E-01	-3.479311804202E-01	5.845419370440E+00
10	238	SR	1.839196975150E-01	-3.479311804202E-01	-5.845419370440E+00
11	9	F	-1.878771454857E+00	-3.333572433911E-01	4.383044839791E+00
12	9	F	-1.878771454857E+00	-3.333572433911E-01	-4.383044839791E+00
13	9	F	1.683121910861E-01	1.718829945061E+00	4.384120846256E+00
14	9	F	1.683121910861E-01	1.718829945061E+00	-4.384120846256E+00
15	238	SR	-1.896746616440E+00	1.736059847794E+00	2.922382182897E+00
16	238	SR	-1.896746616440E+00	1.736059847794E+00	-2.922382182897E+00
17	9	F	1.680267768859E-01	1.718573857244E+00	1.460018816672E+00
18	9	F	1.680267768859E-01	1.718573857244E+00	-1.460018816672E+00
19	9	F	-1.878749000336E+00	-3.328981645705E-01	1.462063600945E+00
20	9	F	-1.878749000336E+00	-3.328981645705E-01	-1.462063600945E+00
21	238	SR	1.854471762091E-01	-3.501984353670E-01	0.000000000000E+00

22	109 F	1.666605389058E+00	-2.203970005955E-01	1.012802649219E+01
23	109 F	1.666605389058E+00	-2.203970005955E-01	-1.012802649219E+01
24	1 H	7.466037789832E-01	-9.054899677088E-01	1.026454855101E+01
25	1 H	7.466037789832E-01	-9.054899677088E-01	-1.026454855101E+01

(100) surface, 50% coverage

1	9 F	-1.049817264200E+00	-1.650797494109E-01	9.954726412549E+00
2	9 F	-1.049817264200E+00	-1.650797494109E-01	-9.954726412549E+00
3	9 F	-4.028400953353E+00	9.815020157379E-01	9.688672822697E+00
4	9 F	-4.028400953353E+00	9.815020157379E-01	-9.688672822697E+00
5	238 SR	2.298322826420E+00	1.920913651168E+00	8.748811658960E+00
6	238 SR	2.298322826420E+00	1.920913651168E+00	-8.748811658960E+00
7	238 SR	-2.087864456132E+00	1.919211667560E+00	8.747191598466E+00
8	238 SR	-2.087864456132E+00	1.919211667560E+00	-8.747191598466E+00
9	9 F	1.047219499822E-01	1.942732685330E+00	7.470184510838E+00
10	9 F	1.047219499822E-01	1.942732685330E+00	-7.470184510838E+00
11	9 F	-4.030245774073E+00	2.045338832487E+00	7.174752685422E+00
12	9 F	-4.030245774073E+00	2.045338832487E+00	-7.174752685422E+00
13	9 F	2.212024731651E+00	-1.019527193930E-01	7.282078661573E+00
14	9 F	2.212024731651E+00	-1.019527193930E-01	-7.282078661573E+00
15	9 F	-2.004619109879E+00	-1.047185672337E-01	7.280206945884E+00
16	9 F	-2.004619109879E+00	-1.047185672337E-01	-7.280206945884E+00
17	238 SR	1.058707848037E-01	-1.030946889478E-01	5.908836322761E+00
18	238 SR	1.058707848037E-01	-1.030946889478E-01	-5.908836322761E+00
19	238 SR	-4.029104605303E+00	-5.198865585881E-02	5.785953038879E+00
20	238 SR	-4.029104605303E+00	-5.198865585881E-02	-5.785953038879E+00
21	9 F	2.146283297114E+00	-7.330787656247E-02	4.376620782011E+00
22	9 F	2.146283297114E+00	-7.330787656247E-02	-4.376620782011E+00
23	9 F	-1.937316277511E+00	-7.244741752878E-02	4.378178725321E+00
24	9 F	-1.937316277511E+00	-7.244741752878E-02	-4.378178725321E+00
25	9 F	1.048530203180E-01	1.978883558445E+00	4.460786837547E+00
26	9 F	1.048530203180E-01	1.978883558445E+00	-4.460786837547E+00
27	9 F	-4.029081720905E+00	2.005893136852E+00	4.324503798851E+00
28	9 F	-4.029081720905E+00	2.005893136852E+00	-4.324503798851E+00
29	238 SR	2.159746693292E+00	1.991919142174E+00	2.921137744225E+00
30	238 SR	2.159746693292E+00	1.991919142174E+00	-2.921137744225E+00
31	238 SR	-1.950654511401E+00	1.994857066769E+00	2.924305489949E+00
32	238 SR	-1.950654511401E+00	1.994857066769E+00	-2.924305489949E+00
33	9 F	1.039451336032E-01	1.994095115570E+00	1.468801923260E+00
34	9 F	1.039451336032E-01	1.994095115570E+00	-1.468801923260E+00
35	9 F	-4.027856124758E+00	1.991790173531E+00	1.456437144811E+00
36	9 F	-4.027856124758E+00	1.991790173531E+00	-1.456437144811E+00
37	9 F	2.165380634054E+00	-7.390115486362E-02	1.458969709155E+00
38	9 F	2.165380634054E+00	-7.390115486362E-02	-1.458969709155E+00
39	9 F	-1.956264776473E+00	-7.273703740438E-02	1.460500377484E+00
40	9 F	-1.956264776473E+00	-7.273703740438E-02	-1.460500377484E+00
41	238 SR	1.033011839527E-01	-7.296763312620E-02	0.000000000000E+00
42	238 SR	-4.026998497965E+00	-7.444446822283E-02	0.000000000000E+00
43	109 F	1.265700838683E+00	-1.666662891397E-01	9.961590964593E+00
44	109 F	1.265700838683E+00	-1.666662891397E-01	-9.961590964593E+00

45	1	H	1.077182408945E-01	-1.682281267100E-01	9.999669490545E+00
46	1	H	1.077182408945E-01	-1.682281267100E-01	-9.999669490545E+00

(100) surface, 25% coverage

1	9	F	5.454421236334E-02	3.199980112350E+00	9.584682513208E+00
2	9	F	5.454421236334E-02	3.199980112350E+00	-9.584682513208E+00
3	9	F	-1.102467844066E+00	-1.621000242378E-01	9.992793810452E+00
4	9	F	-1.102467844066E+00	-1.621000242378E-01	-9.992793810452E+00
5	9	F	-4.078436770695E+00	2.494074482815E+00	9.818089005248E+00
6	9	F	-4.078436770695E+00	2.494074482815E+00	-9.818089005248E+00
7	9	F	-4.077567644694E+00	-1.947942440879E+00	9.930553379335E+00
8	9	F	-4.077567644694E+00	-1.947942440879E+00	-9.930553379335E+00
9	238	SR	2.090242608742E+00	2.004675481096E+00	8.797019228036E+00
10	238	SR	2.090242608742E+00	2.004675481096E+00	-8.797019228036E+00
11	238	SR	2.233075305019E+00	-2.109537667247E+00	8.705657480934E+00
12	238	SR	2.233075305019E+00	-2.109537667247E+00	-8.705657480934E+00
13	238	SR	-1.981320513047E+00	2.004311394989E+00	8.796561071937E+00
14	238	SR	-1.981320513047E+00	2.004311394989E+00	-8.796561071937E+00
15	238	SR	-2.122795292718E+00	-2.110254253855E+00	8.705219587890E+00
16	238	SR	-2.122795292718E+00	-2.110254253855E+00	-8.705219587890E+00
17	9	F	5.478011619649E-02	1.880586865429E+00	7.198833034307E+00
18	9	F	5.478011619649E-02	1.880586865429E+00	-7.198833034307E+00
19	9	F	5.541536116979E-02	-2.158750640779E+00	7.444872944844E+00
20	9	F	5.541536116979E-02	-2.158750640779E+00	-7.444872944844E+00
21	9	F	-4.077254151695E+00	1.931263346542E+00	7.153787756231E+00
22	9	F	-4.077254151695E+00	1.931263346542E+00	-7.153787756231E+00
23	9	F	-4.078544698389E+00	-2.170841733157E+00	7.171663587693E+00
24	9	F	-4.078544698389E+00	-2.170841733157E+00	-7.171663587693E+00
25	9	F	2.177121264904E+00	-1.061305880343E-01	7.324210737708E+00
26	9	F	2.177121264904E+00	-1.061305880343E-01	-7.324210737708E+00
27	9	F	2.163231948348E+00	4.065613465143E+00	7.410105488650E+00
28	9	F	2.163231948348E+00	4.065613465143E+00	-7.410105488650E+00
29	9	F	-2.067215579517E+00	-1.071669037948E-01	7.323159642355E+00
30	9	F	-2.067215579517E+00	-1.071669037948E-01	-7.323159642355E+00
31	9	F	-2.053062055797E+00	4.065631804315E+00	7.409378450412E+00
32	9	F	-2.053062055797E+00	4.065631804315E+00	-7.409378450412E+00
33	238	SR	5.510284722403E-02	-1.284519400265E-01	5.852163596466E+00
34	238	SR	5.510284722403E-02	-1.284519400265E-01	-5.852163596466E+00
35	238	SR	5.490347222950E-02	3.998350824762E+00	5.924407825727E+00
36	238	SR	5.490347222949E-02	3.998350824762E+00	-5.924407825727E+00
37	238	SR	-4.077966673107E+00	-8.944894492205E-02	5.783434997412E+00
38	238	SR	-4.077966673107E+00	-8.944894492205E-02	-5.783434997412E+00
39	238	SR	-4.078395192484E+00	3.990236607028E+00	5.828458491541E+00
40	238	SR	-4.078395192484E+00	3.990236607028E+00	-5.828458491541E+00
41	9	F	2.102520804187E+00	-1.280767468963E-01	4.380869846944E+00
42	9	F	2.102520804187E+00	-1.280767468963E-01	-4.380869846944E+00
43	9	F	2.103069180328E+00	4.002301032877E+00	4.432986538652E+00
44	9	F	2.103069180328E+00	4.002301032877E+00	-4.432986538652E+00
45	9	F	-1.992811758997E+00	-1.278695264454E-01	4.380917785945E+00
46	9	F	-1.992811758997E+00	-1.278695264454E-01	-4.380917785945E+00

47	9	F	-1.993725712236E+00	4.002257523461E+00	4.433112089103E+00
48	9	F	-1.993725712236E+00	4.002257523461E+00	-4.433112089103E+00
49	9	F	5.488209154700E-02	1.948853351718E+00	4.369398973991E+00
50	9	F	5.488209154700E-02	1.948853351718E+00	-4.369398973991E+00
51	9	F	5.460726994478E-02	-2.212672682829E+00	4.438063565080E+00
52	9	F	5.460726994477E-02	-2.212672682829E+00	-4.438063565080E+00
53	9	F	-4.078445598101E+00	1.953362038822E+00	4.307184954773E+00
54	9	F	-4.078445598101E+00	1.953362038822E+00	-4.307184954773E+00
55	9	F	-4.078376772027E+00	-2.199598416541E+00	4.335836056900E+00
56	9	F	-4.078376772027E+00	-2.199598416541E+00	-4.335836056900E+00
57	238	SR	2.109648834844E+00	1.943709326492E+00	2.914460457739E+00
58	238	SR	2.109648834844E+00	1.943709326492E+00	-2.914460457739E+00
59	238	SR	2.115700821867E+00	-2.200798476314E+00	2.930315634187E+00
60	238	SR	2.115700821867E+00	-2.200798476314E+00	-2.930315634187E+00
61	238	SR	-2.000253542070E+00	1.943802818372E+00	2.914942364169E+00
62	238	SR	-2.000253542070E+00	1.943802818372E+00	-2.914942364169E+00
63	238	SR	-2.006917620886E+00	-2.201022463412E+00	2.930701911579E+00
64	238	SR	-2.006917620886E+00	-2.201022463412E+00	-2.930701911579E+00
65	9	F	5.450588321454E-02	1.940379704141E+00	1.447780312718E+00
66	9	F	5.450588321454E-02	1.940379704141E+00	-1.447780312718E+00
67	9	F	5.437792777224E-02	-2.197079858210E+00	1.471063002189E+00
68	9	F	5.437792777224E-02	-2.197079858210E+00	-1.471063002189E+00
69	9	F	-4.078367988631E+00	1.939597246966E+00	1.446280113696E+00
70	9	F	-4.078367988631E+00	1.939597246966E+00	-1.446280113696E+00
71	9	F	-4.078469211674E+00	-2.198441165781E+00	1.458371602180E+00
72	9	F	-4.078469211674E+00	-2.198441165781E+00	-1.458371602180E+00
73	9	F	2.117051872637E+00	-1.326981752270E-01	1.464677166365E+00
74	9	F	2.117051872637E+00	-1.326981752270E-01	-1.464677166365E+00
75	9	F	2.117352655360E+00	4.008308383281E+00	1.468380622260E+00
76	9	F	2.117352655360E+00	4.008308383281E+00	-1.468380622260E+00
77	9	F	-2.008182917661E+00	-1.327074116370E-01	1.464859340116E+00
78	9	F	-2.008182917661E+00	-1.327074116370E-01	-1.464859340116E+00
79	9	F	-2.008004834648E+00	4.008304283817E+00	1.468546289397E+00
80	9	F	-2.008004834648E+00	4.008304283817E+00	-1.468546289397E+00
81	238	SR	5.398943507733E-02	-1.319788397406E-01	0.000000000000E+00
82	238	SR	5.466949694039E-02	4.008568733090E+00	0.000000000000E+00
83	238	SR	-4.078484119899E+00	-1.311129026505E-01	0.000000000000E+00
84	238	SR	-4.078166104932E+00	4.005613688050E+00	0.000000000000E+00
85	109	F	1.214185302695E+00	-1.613912087229E-01	9.995533440803E+00
86	109	F	1.214185302695E+00	-1.613912087229E-01	-9.995533440803E+00
87	1	H	5.603691055607E-02	-1.466989087891E-01	1.004756870274E+01
88	1	H	5.603691055606E-02	-1.466989087891E-01	-1.004756870274E+01

3. BaF2:

(111) surface, 100% coverage

1	9	F	-1.100851029793E+00	1.968033605379E+00	2.590045034633E+00
2	9	F	-1.105322127507E+00	1.853170393361E+00	-2.590045034633E+00
3	256	BA	-1.101707728632E+00	-6.573687137988E-01	1.797979677704E+00

4	256 BA	1.101707728632E+00	6.573687137979E-01	-1.797979677704E+00
5	9 F	1.104312026284E+00	6.391801192552E-01	8.683569029906E-01
6	9 F	-1.104312026284E+00	-6.391801192543E-01	-8.683569029906E-01
7	109 F	-1.179407931282E+00	3.542903644477E-01	4.391299057898E+00
8	1 H	-1.142826164168E+00	1.200269061399E+00	3.878280462758E+00
9	109 F	1.179407931282E+00	-3.542903644477E-01	-4.391299057898E+00
10	1 H	1.142826164168E+00	-1.200269061399E+00	-3.878280462758E+00

(111) surface, 50% coverage

1	9 F	1.037524630547E-01	-2.980057425011E-02	2.688755388173E+00
2	9 F	4.392807107747E+00	-2.110687711068E-03	2.463944996884E+00
3	9 F	-1.037524630547E-01	2.980057425103E-02	-2.688755388173E+00
4	9 F	4.431885521452E+00	2.110687711991E-03	-2.463944996884E+00
5	256 BA	-2.259172144309E+00	1.272793968190E+00	1.776384365513E+00
6	256 BA	2.237910500168E+00	1.291529221215E+00	1.799487086564E+00
7	256 BA	2.259172144309E+00	-1.272793968191E+00	-1.776384365513E+00
8	256 BA	-2.237910500168E+00	-1.291529221216E+00	-1.799487086564E+00
9	9 F	2.183364325081E+00	-1.304324558510E+00	8.774086510950E-01
10	9 F	-2.175503095547E+00	-1.246178488840E+00	8.438395247291E-01
11	9 F	-2.183364325081E+00	1.304324558511E+00	-8.774086510950E-01
12	9 F	2.175503095547E+00	1.246178488841E+00	-8.438395247291E-01
13	109 F	-1.426912120492E+00	6.384369779164E-01	4.390402140772E+00
14	1 H	-6.623744869218E-01	2.871589539620E-01	3.836965450203E+00
15	109 F	1.426912120492E+00	-6.384369779164E-01	-4.390402140772E+00
16	1 H	6.623744869218E-01	-2.871589539620E-01	-3.836965450203E+00

(111) surface, 25% coverage

1	9 F	1.804284958352E-01	-1.039898408531E-01	2.784216664113E+00
2	9 F	-2.228089204028E+00	3.849822958116E+00	2.528946116030E+00
3	9 F	4.376374427994E+00	4.868998145475E-03	2.529241462721E+00
4	9 F	2.225599862600E+00	3.810197960955E+00	2.531942739322E+00
5	9 F	-1.804284958353E-01	1.039898408540E-01	-2.784216664113E+00
6	9 F	-2.184257110572E+00	3.792585039364E+00	-2.528946116030E+00
7	9 F	4.448318201206E+00	-4.868998144552E-03	-2.529241462721E+00
8	9 F	2.186746452000E+00	3.832210036525E+00	-2.531942739322E+00
9	256 BA	-2.279228099268E+00	1.315772012192E+00	1.815227162144E+00
10	256 BA	8.970002063244E-03	-2.585176720610E+00	1.740062068210E+00
11	256 BA	2.243103979112E+00	1.284718576503E+00	1.739842304601E+00
12	256 BA	4.412267353292E+00	-2.547303666085E+00	1.842880075058E+00
13	256 BA	-8.970002063269E-03	2.585176720609E+00	-1.740062068210E+00
14	256 BA	2.279228099268E+00	-1.315772012193E+00	-1.815227162144E+00
15	256 BA	-4.412267353292E+00	2.547303666084E+00	-1.842880075058E+00
16	256 BA	-2.243103979112E+00	-1.284718576504E+00	-1.739842304601E+00
17	9 F	-9.575285525426E-03	2.496497930106E+00	8.984832976719E-01
18	9 F	2.194465635055E+00	-1.266850044744E+00	8.332679671283E-01
19	9 F	-4.421171338974E+00	2.552570720565E+00	7.969940471443E-01
20	9 F	-2.166584565134E+00	-1.239951569455E+00	8.983754139478E-01
21	9 F	-2.194465635055E+00	1.266850044745E+00	-8.332679671283E-01

22	9	F	9.575285525452E-03	-2.496497930105E+00	-8.984832976719E-01
23	9	F	2.166584565134E+00	1.239951569456E+00	-8.983754139478E-01
24	9	F	4.421171338974E+00	-2.552570720564E+00	-7.969940471443E-01
25	109	F	-1.321076237587E+00	7.581047343251E-01	4.399430760729E+00
26	1	H	-5.846862068058E-01	3.350133730440E-01	3.831858528597E+00
27	109	F	1.321076237587E+00	-7.581047343251E-01	-4.399430760729E+00
28	1	H	5.846862068058E-01	-3.350133730440E-01	-3.831858528597E+00

(110) surface, 100% coverage

1	256	BA	1.123043445416E+00	1.560000000000E+00	5.340088156081E+00
2	256	BA	-1.123043445416E+00	-1.560000000000E+00	-5.340088156081E+00
3	9	F	-1.106414689585E+00	2.971246809785E+00	5.449193386137E+00
4	9	F	1.106414689585E+00	-1.487531902150E-01	-5.449193386137E+00
5	9	F	1.106414689585E+00	-2.971246809785E+00	-5.449193386137E+00
6	9	F	-1.106414689585E+00	1.487531902150E-01	5.449193386137E+00
7	256	BA	-1.099645546041E+00	-1.560000000000E+00	3.379306677028E+00
8	256	BA	1.099645546041E+00	1.560000000000E+00	-3.379306677028E+00
9	9	F	1.103937955245E+00	-6.799517556000E-03	3.274679071527E+00
10	9	F	-1.103937955245E+00	3.113200482444E+00	-3.274679071527E+00
11	9	F	-1.103937955245E+00	6.799517556000E-03	-3.274679071527E+00
12	9	F	1.103937955245E+00	-3.113200482444E+00	3.274679071527E+00
13	256	BA	1.103682149579E+00	1.560000000000E+00	1.063269877411E+00
14	256	BA	-1.103682149579E+00	-1.560000000000E+00	-1.063269877411E+00
15	9	F	-1.102598918396E+00	-3.119157938471E+00	1.120057930961E+00
16	9	F	1.102598918396E+00	8.420615290001E-04	-1.120057930961E+00
17	9	F	1.102598918396E+00	3.119157938471E+00	-1.120057930961E+00
18	9	F	-1.102598918396E+00	-8.420615290001E-04	1.120057930961E+00
19	109	F	-1.412631113830E-01	1.560000000000E+00	7.888107155958E+00
20	109	F	1.412631113830E-01	-1.560000000000E+00	-7.888107155958E+00
21	1	H	-8.565441538480E-01	1.560000000000E+00	7.255776793933E+00
22	1	H	8.565441538480E-01	-1.560000000000E+00	-7.255776793933E+00

(110) surface, 50% coverage

1	256	BA	2.198796035955E+00	3.198697006867E+00	5.444653609366E+00
2	256	BA	-2.350914035533E+00	3.175154488080E+00	5.370706930429E+00
3	256	BA	-2.202042507059E-01	8.129407982021E-02	-5.447297864787E+00
4	256	BA	4.335499690927E+00	5.452435971301E-02	-5.369931010326E+00
5	9	F	-8.650882512439E-02	-1.815076392668E+00	5.275900044116E+00
6	9	F	4.331290211856E+00	-1.604040736728E+00	5.445501882863E+00
7	9	F	2.068420407171E+00	1.293226648499E+00	-5.268070912655E+00
8	9	F	-2.349342579077E+00	1.519234292597E+00	-5.449003180586E+00
9	9	F	2.350338553303E+00	-1.726506385395E+00	-5.631741809687E+00
10	9	F	-2.356907575757E+00	-1.387170669468E+00	-5.361163799766E+00
11	9	F	-3.437305830413E-01	1.413436120400E+00	5.620609682799E+00
12	9	F	4.341159552758E+00	1.731964622441E+00	5.363606089191E+00
13	256	BA	-1.088383231498E-01	-2.946462514503E-02	3.326372715433E+00
14	256	BA	4.305276578682E+00	4.621347264315E-02	3.368686628677E+00
15	256	BA	2.093304112565E+00	3.089282905387E+00	-3.327448370085E+00

16	256	BA	-2.321661737685E+00	3.166742207605E+00	-3.368425859072E+00
17	9	F	2.033359894047E+00	1.611488641217E+00	3.368899675221E+00
18	9	F	-2.271112081625E+00	1.604740082065E+00	3.286353800282E+00
19	9	F	-4.614371690151E-02	-1.507245992341E+00	-3.373587699805E+00
20	9	F	4.255167782186E+00	-1.514648383288E+00	-3.284548194267E+00
21	9	F	-1.542908306646E-01	1.597876936053E+00	-3.280049379922E+00
22	9	F	4.326521329386E+00	1.586127535537E+00	-3.267878290453E+00
23	9	F	2.136244299174E+00	-1.523542960126E+00	3.279580462918E+00
24	9	F	-2.340844202883E+00	-1.534533658207E+00	3.268423655524E+00
25	256	BA	2.093420739578E+00	3.132675909657E+00	1.096826330142E+00
26	256	BA	-2.315840757675E+00	3.129396177947E+00	1.061743286323E+00
27	256	BA	-1.089048951433E-01	1.272435699212E-02	-1.098503238443E+00
28	256	BA	4.300200205230E+00	9.633059474101E-03	-1.061647254101E+00
29	9	F	-1.132982529764E-01	-1.555145100823E+00	1.103845738648E+00
30	9	F	4.307084985506E+00	-1.545595327622E+00	1.098413280916E+00
31	9	F	2.097864655482E+00	1.564136790646E+00	-1.104969027536E+00
32	9	F	-2.322343058481E+00	1.573795768711E+00	-1.098112092075E+00
33	9	F	2.083665422007E+00	-1.563721150958E+00	-1.098360094508E+00
34	9	F	-2.303691066457E+00	-1.540773569220E+00	-1.132135281894E+00
35	9	F	-9.942036319855E-02	1.557248766472E+00	1.097100319478E+00
36	9	F	4.288820645182E+00	1.579608953056E+00	1.131650947375E+00
37	109	F	1.126116400864E+00	1.618156456783E+00	7.438786006610E+00
38	1	H	4.076125759811E-01	1.421636475491E+00	6.704069078349E+00
39	109	F	8.593852928759E-01	-1.499118990591E+00	-7.428257394221E+00
40	1	H	1.585564596766E+00	-1.702432171575E+00	-6.700599410472E+00

(110) surface, 25% coverage

1	256	BA	2.228145984543E+00	3.162233389399E+00	5.430216756431E+00
2	256	BA	2.151049149818E+00	-3.111805378127E+00	5.361100721464E+00
3	256	BA	-2.301796265647E+00	3.158685979471E+00	5.373069575193E+00
4	256	BA	-2.262564763697E+00	-3.119669982118E+00	5.349450222108E+00
5	256	BA	-1.401470074935E-01	4.694015030061E-02	-5.433906534339E+00
6	256	BA	-6.118808803541E-02	-6.235650155417E+00	-5.356494661307E+00
7	256	BA	4.391890042581E+00	4.202622002905E-02	-5.367825560203E+00
8	256	BA	4.352049851185E+00	6.236179695411E+00	-5.353654437620E+00
9	9	F	-4.653003796231E-02	4.469496373932E+00	5.360738556810E+00
10	9	F	-5.775620328966E-02	-1.697402955437E+00	5.372148248810E+00
11	9	F	4.369655411500E+00	4.607077115611E+00	5.477680813654E+00
12	9	F	4.359458094616E+00	-1.674528774394E+00	5.414502648093E+00
13	9	F	2.135836732483E+00	1.349384527713E+00	-5.350334183243E+00
14	9	F	2.146967833327E+00	-4.820768300575E+00	-5.379871327110E+00
15	9	F	-2.280487820943E+00	1.490993286440E+00	-5.473802183183E+00
16	9	F	-2.269564039777E+00	-4.797322113069E+00	-5.419954398253E+00
17	9	F	2.313169552565E+00	-1.639624478456E+00	-5.612539460876E+00
18	9	F	2.144198379458E+00	4.765868035040E+00	-5.442254052723E+00
19	9	F	-2.295758160920E+00	-1.415476623125E+00	-5.391098619917E+00
20	9	F	-2.266121470332E+00	4.797572719098E+00	-5.421928369785E+00
21	9	F	-2.153706441771E-01	1.481666500329E+00	5.601669424619E+00
22	9	F	-5.525539403897E-02	-4.589869502996E+00	5.448996802808E+00
23	9	F	4.385391917971E+00	1.701539841989E+00	5.388035864915E+00

24	9	F	4.356713260274E+00	-4.559799054434E+00	5.427549894776E+00
25	256	BA	-6.395413550518E-02	-3.621183557939E-02	3.328908807190E+00
26	256	BA	-4.901870503561E-02	6.236090674723E+00	3.378673705886E+00
27	256	BA	4.352385563638E+00	4.194132684551E-03	3.381960362666E+00
28	256	BA	4.356861656440E+00	-6.216729943557E+00	3.369238120858E+00
29	256	BA	2.138502829548E+00	3.115751558562E+00	-3.366663974946E+00
30	256	BA	2.154211840915E+00	-3.156394129164E+00	-3.341026841360E+00
31	256	BA	-2.267143742317E+00	3.143929095429E+00	-3.363165719970E+00
32	256	BA	-2.262340557724E+00	-3.116996385870E+00	-3.387661477308E+00
33	9	F	2.094882550615E+00	1.580898479713E+00	3.359722477629E+00
34	9	F	2.146195433904E+00	-4.664048962779E+00	3.287361387774E+00
35	9	F	-2.228076192619E+00	1.581371635382E+00	3.305731606169E+00
36	9	F	-2.254151693102E+00	-4.668660543770E+00	3.278051107098E+00
37	9	F	-4.125122591711E-03	-1.537678623407E+00	-3.368064131220E+00
38	9	F	-5.559545081311E-02	4.694363580704E+00	-3.279724077754E+00
39	9	F	4.319145833345E+00	-1.536053814340E+00	-3.303396497328E+00
40	9	F	4.343014256348E+00	4.688540829207E+00	-3.278981766383E+00
41	9	F	-8.959266934354E-02	1.569904160036E+00	-3.301828366189E+00
42	9	F	-6.210701075006E-02	-4.681250029770E+00	-3.271279969447E+00
43	9	F	4.370852829626E+00	1.569376052384E+00	-3.285462382216E+00
44	9	F	4.362385308196E+00	-4.686234813510E+00	-3.273937399959E+00
45	9	F	2.177918436900E+00	4.688640907859E+00	3.304164055146E+00
46	9	F	2.152900211860E+00	-1.560230753100E+00	3.270635204161E+00
47	9	F	-2.280528145652E+00	4.687581738232E+00	3.293699588457E+00
48	9	F	-2.272547913410E+00	-1.564118722764E+00	3.265983510838E+00
49	256	BA	2.144962495961E+00	3.118602085516E+00	1.093513520532E+00
50	256	BA	2.151023758800E+00	-3.120425683998E+00	1.067067356894E+00
51	256	BA	-2.263028822570E+00	3.117273510443E+00	1.076809787592E+00
52	256	BA	-2.265499830653E+00	-3.121574430724E+00	1.052793739324E+00
53	256	BA	-5.550990537529E-02	3.465178584389E-03	-1.101769506436E+00
54	256	BA	-5.987084007267E-02	6.234612820341E+00	-1.057588525601E+00
55	256	BA	4.352519016374E+00	5.089711708481E-03	-1.069199523938E+00
56	256	BA	4.355761204644E+00	6.230578263000E+00	-1.060406063996E+00
57	9	F	-6.050308141756E-02	-1.561592072432E+00	1.095006903508E+00
58	9	F	-5.836487388004E-02	4.672917019886E+00	1.129477683929E+00
59	9	F	4.356936144942E+00	-1.558326202915E+00	1.102175895341E+00
60	9	F	4.356052175822E+00	4.675074746605E+00	1.119692351290E+00
61	9	F	2.148404297333E+00	1.558489505166E+00	-1.110545024833E+00
62	9	F	2.150757462391E+00	-4.687220992345E+00	-1.113278795607E+00
63	9	F	-2.267994247012E+00	1.559948299707E+00	-1.105920849194E+00
64	9	F	-2.264936345288E+00	-4.683840494692E+00	-1.115485685007E+00
65	9	F	2.144005593975E+00	4.672279139829E+00	-1.097222196909E+00
66	9	F	2.143071525830E+00	-1.565712734127E+00	-1.117739341194E+00
67	9	F	-2.262610717595E+00	4.679601657951E+00	-1.104135463856E+00
68	9	F	-2.255850282416E+00	-1.556642563220E+00	-1.143885290048E+00
69	9	F	-5.347839051291E-02	1.547215485403E+00	1.100837446418E+00
70	9	F	-5.394928634786E-02	-4.679715571329E+00	1.114727792180E+00
71	9	F	4.345345274427E+00	1.559245636467E+00	1.136242636422E+00
72	9	F	4.353635135724E+00	-4.676241253911E+00	1.111801671827E+00
73	109	F	1.073179839759E+00	1.738161903348E+00	7.551103986368E+00
74	1	H	4.291384023987E-01	1.509914233905E+00	6.762993562791E+00
75	109	F	1.014787234302E+00	-1.370896455363E+00	-7.553026877568E+00

76	1	H	1.662188996778E+00	-1.604061546720E+00	-6.768474261142E+00

(100) surface, 100% coverage

1	9	F	5.064933075265E-02	1.113070620279E+00	1.070494692967E+01
2	9	F	5.064933075264E-02	1.113070620279E+00	-1.070494692967E+01
3	256	BA	-2.028866473901E+00	1.855008920421E+00	9.346425784753E+00
4	256	BA	-2.028866473901E+00	1.855008920421E+00	-9.346425784753E+00
5	9	F	1.896220438966E-01	1.899387736260E+00	7.781555695078E+00
6	9	F	1.896220438966E-01	1.899387736260E+00	-7.781555695078E+00
7	9	F	-1.992313772555E+00	-3.367632422844E-01	7.788833960868E+00
8	9	F	-1.992313772555E+00	-3.367632422844E-01	-7.788833960868E+00
9	256	BA	2.061021424218E-01	-3.155803893604E-01	6.233104500177E+00
10	256	BA	2.061021424218E-01	-3.155803893604E-01	-6.233104500177E+00
11	9	F	-1.998571639680E+00	-3.124992599877E-01	4.675552931839E+00
12	9	F	-1.998571639680E+00	-3.124992599877E-01	-4.675552931839E+00
13	9	F	2.079516475765E-01	1.892833673603E+00	4.673133149700E+00
14	9	F	2.079516475765E-01	1.892833673603E+00	-4.673133149700E+00
15	256	BA	-1.998528223978E+00	1.893575626654E+00	3.116123542333E+00
16	256	BA	-1.998528223978E+00	1.893575626654E+00	-3.116123542333E+00
17	9	F	2.075466057193E-01	1.893937904450E+00	1.557731374621E+00
18	9	F	2.075466057192E-01	1.893937904450E+00	-1.557731374621E+00
19	9	F	-1.998537638644E+00	-3.125114986059E-01	1.558354182157E+00
20	9	F	-1.998537638644E+00	-3.125114986059E-01	-1.558354182157E+00
21	256	BA	2.075929438837E-01	-3.123414775095E-01	0.000000000000E+00
22	109	F	1.673494011396E+00	-4.898018934712E-01	1.075111421098E+01
23	109	F	1.673494011396E+00	-4.898018934712E-01	-1.075111421098E+01
24	1	H	7.667897085580E-01	2.078588554013E-01	1.084395158471E+01
25	1	H	7.667897085580E-01	2.078588554013E-01	-1.084395158471E+01

(100) surface, 50% coverage

1	9	F	-1.054252235311E+00	7.805383644079E-02	1.055465881159E+01
2	9	F	-1.054252235311E+00	7.805383644079E-02	-1.055465881159E+01
3	9	F	-4.306506739701E+00	1.068544071701E+00	1.014993897612E+01
4	9	F	-4.306506739701E+00	1.068544071701E+00	-1.014993897612E+01
5	256	BA	-2.233265243738E+00	-2.128037168198E+00	9.315162077010E+00
6	256	BA	-2.233265243738E+00	-2.128037168198E+00	-9.315162077010E+00
7	256	BA	2.445772684330E+00	2.285610916061E+00	9.316312869656E+00
8	256	BA	2.445772684330E+00	2.285610916061E+00	-9.316312869656E+00
9	9	F	1.074521490872E-01	-2.103983302322E+00	7.928395030050E+00
10	9	F	1.074521490872E-01	-2.103983302322E+00	-7.928395030050E+00
11	9	F	-4.305860980127E+00	-1.973450778819E+00	7.667937609231E+00
12	9	F	-4.305860980127E+00	-1.973450778819E+00	-7.667937609231E+00
13	9	F	-2.134547788703E+00	1.300309608740E-01	7.757066137882E+00
14	9	F	-2.134547788703E+00	1.300309608740E-01	-7.757066137882E+00
15	9	F	2.348223610428E+00	1.305003138832E-01	7.760352423027E+00
16	9	F	2.348223610428E+00	1.305003138832E-01	-7.760352423027E+00
17	256	BA	1.059664772940E-01	1.251175989105E-01	6.290982141514E+00
18	256	BA	1.059664772940E-01	1.251175989105E-01	-6.290982141514E+00

19	256	BA	-4.306525960076E+00	1.881120792807E-01	6.179266353554E+00
20	256	BA	-4.306525960076E+00	1.881120792807E-01	-6.179266353554E+00
21	9	F	-2.074985132133E+00	1.605480300800E-01	4.668985841257E+00
22	9	F	-2.074985132133E+00	1.605480300800E-01	-4.668985841257E+00
23	9	F	2.287386444602E+00	1.601891918977E-01	4.670151907748E+00
24	9	F	2.287386444602E+00	1.601891918977E-01	-4.670151907748E+00
25	9	F	1.058381818337E-01	-2.066147465674E+00	4.737404675295E+00
26	9	F	1.058381818337E-01	-2.066147465674E+00	-4.737404675295E+00
27	9	F	-4.306279174222E+00	-2.025317678112E+00	4.624718219169E+00
28	9	F	-4.306279174222E+00	-2.025317678112E+00	-4.624718219169E+00
29	256	BA	-2.088001957052E+00	-2.045841638272E+00	3.116000914459E+00
30	256	BA	-2.088001957052E+00	-2.045841638272E+00	-3.116000914459E+00
31	256	BA	2.299466242339E+00	-2.046352787584E+00	3.116802185808E+00
32	256	BA	2.299466242339E+00	-2.046352787584E+00	-3.116802185808E+00
33	9	F	1.059527307742E-01	-2.044179734214E+00	1.560786857295E+00
34	9	F	1.059527307742E-01	-2.044179734214E+00	-1.560786857295E+00
35	9	F	-4.306937875108E+00	-2.048266497013E+00	1.556967886693E+00
36	9	F	-4.306937875108E+00	-2.048266497013E+00	-1.556967886693E+00
37	9	F	-2.095764248119E+00	1.602070490596E-01	1.556908995277E+00
38	9	F	-2.095764248119E+00	1.602070490596E-01	-1.556908995277E+00
39	9	F	2.307032441334E+00	1.599400122093E-01	1.557690642688E+00
40	9	F	2.307032441334E+00	1.599400122093E-01	-1.557690642688E+00
41	256	BA	1.058608241577E-01	1.614944094388E-01	0.000000000000E+00
42	256	BA	-4.306979013011E+00	1.585247574118E-01	0.000000000000E+00
43	109	F	1.265687541350E+00	7.889890657041E-02	1.056075045411E+01
44	109	F	1.265687541350E+00	7.889890657041E-02	-1.056075045411E+01
45	1	H	1.070869149983E-01	7.683251794566E-02	1.058795145598E+01
46	1	H	1.070869149983E-01	7.683251794566E-02	-1.058795145598E+01

(100) surface, 25% coverage

1	9	F	8.326379382651E-01	-4.321203858982E+00	1.007510886827E+01
2	9	F	8.326379382651E-01	-4.321203858982E+00	-1.007510886827E+01
3	9	F	-3.817083055741E-02	1.236990446215E+00	1.055676109652E+01
4	9	F	-3.817083055741E-02	1.236990446215E+00	-1.055676109652E+01
5	9	F	-4.327005521525E+00	-3.106543645182E+00	1.029327153662E+01
6	9	F	-4.327005521525E+00	-3.106543645182E+00	-1.029327153662E+01
7	9	F	4.442323676185E+00	1.683496035560E+00	1.042721874951E+01
8	9	F	4.442323676185E+00	1.683496035560E+00	-1.042721874951E+01
9	256	BA	-2.228165348286E+00	-2.131009012458E+00	9.278232804944E+00
10	256	BA	-2.228165348286E+00	-2.131009012458E+00	-9.278232804944E+00
11	256	BA	-2.241355667189E+00	2.355421177142E+00	9.315096619323E+00
12	256	BA	-2.241355667189E+00	2.355421177142E+00	-9.315096619323E+00
13	256	BA	2.221343303444E+00	-2.288365860475E+00	9.343852139859E+00
14	256	BA	2.221343303444E+00	-2.288365860475E+00	-9.343852139859E+00
15	256	BA	2.228407226704E+00	2.435178883968E+00	9.351168710701E+00
16	256	BA	2.228407226704E+00	2.435178883968E+00	-9.351168710701E+00
17	9	F	-8.422749593717E-03	-2.113034363767E+00	7.733190131287E+00
18	9	F	-8.422749593717E-03	-2.113034363767E+00	-7.733190131287E+00
19	9	F	-4.448219912403E-03	2.328979652902E+00	7.728697597687E+00
20	9	F	-4.448219912403E-03	2.328979652902E+00	-7.728697597687E+00

21	9	F	-4.338485072341E+00	-2.012035233960E+00	7.673766918191E+00
22	9	F	-4.338485072341E+00	-2.012035233960E+00	-7.673766918191E+00
23	9	F	-4.344649164392E+00	2.407718050928E+00	7.660646159934E+00
24	9	F	-4.344649164392E+00	2.407718050928E+00	-7.660646159934E+00
25	9	F	-2.209886139569E+00	1.315840228752E-01	7.967326819790E+00
26	9	F	-2.209886139569E+00	1.315840228752E-01	-7.967326819790E+00
27	9	F	-2.170362754167E+00	-4.290240181648E+00	7.882946590117E+00
28	9	F	-2.170362754167E+00	-4.290240181648E+00	-7.882946590117E+00
29	9	F	2.235212320964E+00	1.049996599428E-01	7.959429174354E+00
30	9	F	2.235212320964E+00	1.049996599428E-01	-7.959429174354E+00
31	9	F	2.343532783934E+00	-4.293715506888E+00	7.663365525406E+00
32	9	F	2.343532783934E+00	-4.293715506888E+00	-7.663365525406E+00
33	256	BA	4.938378328713E-02	1.217619406052E-01	6.288020383074E+00
34	256	BA	4.938378328713E-02	1.217619406052E-01	-6.288020383074E+00
35	256	BA	6.599128519868E-02	-4.298945692234E+00	6.236703724777E+00
36	256	BA	6.599128519868E-02	-4.298945692234E+00	-6.236703724777E+00
37	256	BA	-4.406071139379E+00	1.707550925447E-01	6.248332255002E+00
38	256	BA	-4.406071139379E+00	1.707550925447E-01	-6.248332255002E+00
39	256	BA	-4.347450033485E+00	-4.247469270856E+00	6.168106322111E+00
40	256	BA	-4.347450033485E+00	-4.247469270856E+00	-6.168106322111E+00
41	9	F	-2.161383126520E+00	1.455011566870E-01	4.746711155259E+00
42	9	F	-2.161383126520E+00	1.455011566870E-01	-4.746711155259E+00
43	9	F	-2.127368216161E+00	-4.267979587785E+00	4.680707727114E+00
44	9	F	-2.127368216161E+00	-4.267979587785E+00	-4.680707727114E+00
45	9	F	2.238445192832E+00	1.473546875850E-01	4.726913267120E+00
46	9	F	2.238445192832E+00	1.473546875850E-01	-4.726913267120E+00
47	9	F	2.250637744533E+00	-4.267201916856E+00	4.642802864344E+00
48	9	F	2.250637744533E+00	-4.267201916856E+00	-4.642802864344E+00
49	9	F	5.292394239871E-02	-2.060955774114E+00	4.674818618930E+00
50	9	F	5.292394239871E-02	-2.060955774114E+00	-4.674818618930E+00
51	9	F	5.350901736290E-02	2.322048866340E+00	4.669747011582E+00
52	9	F	5.350901736290E-02	2.322048866340E+00	-4.669747011582E+00
53	9	F	-4.365948179865E+00	-2.031938800465E+00	4.633319190321E+00
54	9	F	-4.365948179865E+00	-2.031938800465E+00	-4.633319190321E+00
55	9	F	-4.365916705035E+00	2.353331203983E+00	4.628924025360E+00
56	9	F	-4.365916705035E+00	2.353331203983E+00	-4.628924025360E+00
57	256	BA	-2.149925947514E+00	-2.052070960216E+00	3.123673838992E+00
58	256	BA	-2.149925947514E+00	-2.052070960216E+00	-3.123673838992E+00
59	256	BA	-2.150360662101E+00	2.341889244442E+00	3.121410711640E+00
60	256	BA	-2.150360662101E+00	2.341889244442E+00	-3.121410711640E+00
61	256	BA	2.249020377954E+00	-2.056085608394E+00	3.111672214695E+00
62	256	BA	2.249020377954E+00	-2.056085608394E+00	-3.111672214695E+00
63	256	BA	2.249627077309E+00	2.347216880245E+00	3.108368540949E+00
64	256	BA	2.249627077309E+00	2.347216880245E+00	-3.108368540949E+00
65	9	F	4.618855638471E-02	-2.056438651943E+00	1.554398831828E+00
66	9	F	4.618855638471E-02	-2.056438651943E+00	-1.554398831828E+00
67	9	F	4.613443999393E-02	2.349910218881E+00	1.552592642721E+00
68	9	F	4.613443999393E-02	2.349910218881E+00	-1.552592642721E+00
69	9	F	-4.359622085969E+00	-2.059968072475E+00	1.554106730727E+00
70	9	F	-4.359622085969E+00	-2.059968072475E+00	-1.554106730727E+00
71	9	F	-4.359466477533E+00	2.347045700955E+00	1.551977710060E+00
72	9	F	-4.359466477533E+00	2.347045700955E+00	-1.551977710060E+00

73	9	F	-2.153018217974E+00	1.445007262911E-01	1.568908945020E+00
74	9	F	-2.153018217974E+00	1.445007262911E-01	-1.568908945020E+00
75	9	F	-2.156230282353E+00	-4.266865087859E+00	1.568902567217E+00
76	9	F	-2.156230282353E+00	-4.266865087859E+00	-1.568902567217E+00
77	9	F	2.254209899147E+00	1.448687199010E-01	1.560089489036E+00
78	9	F	2.254209899147E+00	1.448687199010E-01	-1.560089489036E+00
79	9	F	2.252738472373E+00	-4.266386749776E+00	1.553761386581E+00
80	9	F	2.252738472373E+00	-4.266386749776E+00	-1.553761386581E+00
81	256	BA	4.841910445268E-02	1.458762475021E-01	0.000000000000E+00
82	256	BA	4.480347370047E-02	-4.265439589451E+00	0.000000000000E+00
83	256	BA	-4.360073867015E+00	1.432240771248E-01	0.000000000000E+00
84	256	BA	-4.360139033330E+00	-4.267634999426E+00	0.000000000000E+00
85	109	F	-3.015086988296E-02	-1.080471970212E+00	1.060412121912E+01
86	109	F	-3.015086988296E-02	-1.080471970212E+00	-1.060412121912E+01
87	1	H	-2.574157731560E-02	7.730070120520E-02	1.061230124660E+01
88	1	H	-2.574157731560E-02	7.730070120520E-02	-1.061230124660E+01

P.S. : The format is according to notations in the CRYSTAL09 code.
